# Sugar and low/no-calorie-sweetened beverage consumption and associations with body weight and waist circumference changes in five European cohort studies: the SWEET project

**DOI:** 10.1007/s00394-023-03192-y

**Published:** 2023-07-05

**Authors:** Marion E. C. Buso, Elske M. Brouwer-Brolsma, Novita D. Naomi, Joy Ngo, Sabita S. Soedamah-Muthu, Christina Mavrogianni, Joanne A. Harrold, Jason C. G. Halford, Anne Raben, Johanna M. Geleijnse, Yannis Manios, Luis Serra-Majem, Edith J. M. Feskens

**Affiliations:** 1 Division of Human Nutrition and Health, Wageningen University and Research, BP 17, 6700AA Wageningen, The Netherlands; 2 Nutrition Research Foundation – Fundación para la Investigación Nutricional, Barcelona, Spain; 3 Department of Medical and Clinical Psychology, Center of Research on Psychological and Somatic Disorders (CORPS), Tilburg University, Tilburg, The Netherlands; 4 Institute for Food, Nutrition and Health, University of Reading, Reading, UK; 5 Department of Nutrition and Dietetics, School of Health Science and Education, Harokopio University, Athens, Greece; 6 Institute of Agri-Food and Life Sciences, Hellenic Mediterranean University Research Centre, Heraklion, Greece; 7 Department of Psychology, University of Liverpool, Liverpool, UK; 8 School of Psychology, University of Leeds, Leeds, UK; 9 Department of Nutrition, Exercise, and Sports, Faculty of Science, University of Copenhagen, Copenhagen, Denmark; 10 Clinical Research, Copenhagen University Hospital – Steno Diabetes Center Copenhagen, Herlev, Denmark; 11 Research Institute of Biomedical and Health Sciences (IUIBS), Preventive Medicine Service, Centro Hospitalario Universitario Insular Materno Infantil (CHUIMI), Canarian Health Service, University of Las Palmas de Gran Canaria, Las Palmas, Spain; 12 Centro de Investigación Biomédica en Red Fisiopatologia de la Obesidad y Nutrición (CIBEROBN), Instituto de Salud Carlos III (ISCIII), Madrid, Spain

**Keywords:** Non-nutritive sweeteners, Sugars, Cohort studies, Adults, Weight gain

## Abstract

**Purpose:**

Results of prospective studies investigating associations between low/no-calorie sweeteners (LNCS) and body weight-related outcomes are inconclusive. We conducted dose–response and theoretical replacement individual patient data meta-analyses using harmonised prospective data to evaluate associations between sugar-sweetened beverage (SSB) consumption, low/no-calorie sweetened beverage (LNCB) consumption, and changes in body weight and waist circumference.

**Methods:**

Individual participant data were obtained from five European studies, i.e., Lifelines Cohort Study, NQplus study, Alpha Omega Cohort, Predimed-Plus study, and Feel4diabetes study, including 82,719 adults aged 18–89 with follow-up between 1 and 9 years. Consumption of SSB and LNCB was assessed using food-frequency questionnaires. Multiple regression analyses adjusting for major confounders and including substitution models were conducted to quantify associations in individual cohorts; random-effects meta-analyses were performed to pool individual estimates.

**Results:**

Overall, pooled results showed weak adverse associations between SSB consumption and changes in body weight (+ 0.02 kg/y, 95%CI 0.00; 0.04) and waist circumference (+ 0.03 cm/y, 95%CI 0.01; 0.05). LNCB consumption was associated with higher weight gain (+ 0.06 kg/y, 95%CI 0.04; 0.08) but not with waist circumference. No clear associations were observed for any theoretical replacements, i.e., LNCB or water for SSB or water for LNCB.

**Conclusion:**

In conclusion, this analysis of five European studies found a weak positive association between SSB consumption and weight and waist change, whilst LNCB consumption was associated with weight change only. Theoretical substitutions did not show any clear association. Thus, the benefit of LNCBs as an alternative to SSBs remains unclear.

**Supplementary Information:**

The online version contains supplementary material available at 10.1007/s00394-023-03192-y.

## Introduction

The health effects of low/no-calorie sweeteners (LNCS) have been widely explored, but evidence has been inconclusive. Findings from meta-analyses of prospective studies on low/no-calorie sweetened beverage (LNCB) consumption and body weight development either report no association [1] or adverse associations [2–6]. In contrast, randomised control trials (RCT) suggest beneficial effects of replacing sugar-sweetened beverage (SSB) consumption with LNCB consumption to prevent weight gain [1, 2, 7]. Accordingly, a review of 12 RCTs showed that replacing SSBs with LNCBs resulted in a body weight reduction of 1.06 kg over a median 12-week period [8].

Only a few prospective studies have examined the theoretical replacement of SSBs with LNCBs, and its association with weight measures [9–11], showing either beneficial [9] or no associations [10, 11]. In contrast, we recently observed that the theoretical replacement of SSB consumption with LNCB consumption was associated with higher weight and waist circumference change amongst 78,826 Dutch adults [12]. Thus, despite the low caloric content of LNCBs and its suggested beneficial effects on weight gain based on RCTs, data from prospective studies do not unambiguously support the hypothesis that LNCBs may prevent weight gain.

Various aspects may explain the observed conflicting results on LNCBs and body weight development, including differences in study population, follow-up period, and study design. Whilst RCTs generally focus on acute and shorter-term effects [13–15], observational studies have the potential to explore longer-term associations in an uncontrolled real-life context, although they are more prone to residual confounding [9]. Thus far, various meta-analyses have already been conducted [1–3, 5, 6, 16]; all using summarised data from existing publications that each have inconsistent confounder adjustments, and none of these meta-analyses included theoretical replacement that may be more comparable to the results of intervention studies where LNCBs were a substitute for SSBs or for water.

Considering the above, we used individual participant data that are harmonised across cohorts [17], to study the associations of SSBs and LNCBs with body weight and waist circumference, and theoretical substitution associations between SSBs, LNCBs, and water in five long-term European studies.

## Subjects and methods

### Study population and design

The SWEET project is a Horizon2020 funded project that aims to develop and review evidence on long-term benefits and potential risks involved with replacing sugars with LNCS and sweetness enhancers in the context of public health and safety, obesity, and sustainability (https://sweetproject.eu/). The present study describes longitudinal analyses using data of five studies: the Lifelines Cohort Study (the Netherlands), the Nutrition Questionnaires plus (NQplus) study (the Netherlands), the Alpha Omega Cohort (the Netherlands), the Predimed-Plus cohort (Spain), and the Feel4diabetes study (Greece). An overview of key characteristics of the population studies is available in Suppl. Table 1. All studies were conducted according to the principles of the Declaration of Helsinki and ethical approvals were provided by respective local ethics committees. All participants gave written informed consent before participating. Predimed-Plus was registered with isrctn.com, ISRCTN89898870. The Alpha Omega Cohort and The Feel4Diabetes-study are registered with ClinicalTrials.gov, NCT03192410 and NCT02393872, respectively.

#### The Lifelines Cohort

The Lifelines Cohort Study is a prospective cohort study with a unique three-generation design involving populations in three Northern provinces of the Netherlands (Groningen, Friesland, and Drenthe), including children (0–18 years old), adults (18–65 years old), and older adults (> 65 years old) [18]. Potential participants with severe psychiatric or physical illness, limited life expectancy (< 5 y) or insufficient knowledge of the Dutch language were not eligible for participation. Participants were recruited between 2006 and 2013 and will be followed for over 30 years. Every one and a half years, participants are invited to complete a follow-up questionnaire and on average of every 5 years, several physical measurements are performed and additional questionnaires are administrated. In total, 167,729 participants (inhabitants and their family) from all ages were registered. The Lifelines Cohort Study employs a broad range of investigative procedures in assessing the biomedical, socio-demographic, behavioural, physical and psychological factors which contribute to the health and disease of the general population, with a special focus on multi-morbidities and complex genetics. At the time of the current analysis, baseline data of 152,728 adults (> 18 years old) was available.

#### The Nutrition Questionnaires Plus (NQplus) study

The NQplus study is a longitudinal observational study focussing on dietary assessment and health conducted amongst men and women aged 20–70 years old living in the surroundings of Ede, Wageningen, Renkum, Arnhem, Barneveld, and Veenendaal (The Netherlands) [19, 20]. Recruitment started in June 2011 and ended in February 2013. Participants were followed for 2 years with repeated measurements at 1 year and at the end of the 2 years. In total, 2048 Dutch adults were included and provided a wide range of data resulting from blood and urine analyses (e.g. glucose metabolism), a variety of questionnaires on lifestyle, general health, disease history, physical activity, dietary intakes, food preference and eating behaviour, and physical assessments such as anthropometrics, blood pressure, vascular health and cognitive performance.

#### The Alpha Omega Cohort

The Alpha Omega Cohort consists of 4837 Dutch patients aged 60–80 years with a history of myocardial infarction (MI) up to 10 years before study enrolment [21, 22]. During 40 months of follow-up, patients were randomised to low doses of *n*-3 fatty acids (in margarine) or placebo. This trial phase revealed that administration of low-dose supplementation of the omega-3 fatty acids eicosapentaenoic acid (EPA) and docosahexaenoic acid (DHA) and alpha linolenic acid (ALA) did not reduce cardiovascular events in patients with history of heart attack [22]. At baseline, patients filled in questionnaires and underwent physical examination by trained research nurses, including blood sampling. The dataset comprises data on demographics and socio-economic status, health status, history of diseases, blood biochemistry, lifestyle, physical activity, dietary intakes and anthropometrics. Physical examination was repeated after 40 months in approximately half of the patients who were still alive and who had been enrolled before August 2005. The Alpha Omega Cohort itself now aims to examine predictors of survival in patients with a history of myocardial infarction (post-MI) and includes follow-up for mortality after the 40-month initial period.

#### The Predimed-Plus study

The Predimed-Plus study is a multi-centre RCT, which builds upon the Predimed trial (in Spanish: PREvencion con Dieta MEDiterranea) prevention trial) [23]. The Predimed trial showed that a long-term adherence to an energy-unrestricted Mediterranean diet (MedDiet) supplemented with olive oil or nuts reduced CVD by 30% [24]. As the Predimed trial only focussed on the composition of the diet but not on other lifestyle factors, Predimed-Plus aimed to investigate whether an intentional body mass reduction through the promotion of physical activity and energy restricted MedDiet could decrease the development of cardiovascular disease in the long term [25]. Predimed-Plus was conducted amongst men aged 55–75 years and women aged 60–75 years with a body mass index (BMI) ≥ 27 and < 40 kg/m^2^ fulfilling at least three criteria of the metabolic syndrome at baseline [25]. Recruitment, randomisation (control and intervention stratified by sex, age and centre strata) and baseline measurements were conducted between October 2013 and December 2016 across 23 Spanish study centres, which eventually resulted in the inclusion of a total of 6874 participants. The Predimed-Plus trial will end after a total intervention time of 6 years, but additional follow-up measurements after this date will be conducted for observational purposes. Both the control and intervention group received recommendations to follow the MedDiet. Additionally, the intervention group was assigned to an energy restriction (erMedDiet) with a reduction of about 600 kcal/day and received counselling to increase their physical activity level [25]. Biological samples and anthropometric data were collected as well as information on socio-demographics (age, sex, origin, education, etc.), health, lifestyle (diet, physical activity, smoking, history of diseases and medications), neuropsychological status and quality of life. At the time of the current analysis, follow-up measurements had been conducted at 6 months, 1 year and 2 years after baseline. For the purpose of the SWEET project, prospective data of 266 adults from the Canary Islands study centre were available for the current analysis.

#### The Feel4diabetes study

The Feel4Diabetes study is a EU-funded school and community-based intervention to prevent type-2 diabetes in vulnerable families across Europe by promoting healthy eating and an active lifestyle [26]. It is a cluster-RCT with two components: (1) the ‘all families’ component disseminated via the school setting, and (2) the ‘high-risk families’ component disseminated through community health-care centres. As such, recommendations were provided through supportive social and physical environments at home, school and municipality level, as well as through lifestyle counselling to families with increased risk for type-2 diabetes [26]. Participants were recruited from January to March 2016 within selected provinces in Belgium, Bulgaria, Finland, Greece, Hungary and Spain. Baseline measurements were performed from April to June 2016 and included information on anthropometric indices, blood biochemistry, physical activity and other data such as demographics, socio-economic, lifestyle and health status. The first year intervention period for the high-risk families was conducted between September 2016 and March 2017; the second intervention year started 1 year later. For the purpose of the SWEET project, prospective data of 696 Greek adults/parents were available for the current analysis.

### Anthropometry

In all cohorts, all anthropometric measurements, including body weight and waist circumference were carried out at baseline and follow-up by trained professionals. Body weight was measured with calibrated scales after participants were asked to wear light clothing and remove their shoes. Height and waist circumference were measured with a stadiometer and measuring tape, respectively. BMI was obtained by dividing the weight of participants by the square of their height (kg/m^2^). In all cohorts, weight change (kg/y) and waist circumference change (cm/y) were calculated as: (follow-up measure – baseline measure)/years of follow-up.

### Dietary assessment

All cohorts assessed dietary intake at baseline by means of a Food Frequency Questionnaire (FFQ). In the Lifelines Cohort Study, dietary data were assessed with a 110 item-FFQ [27]. In NQplus, participants completed a validated 183-item semi-quantitative FFQ [28–30]. In both Lifelines and NQplus, average energy and daily intakes were calculated by multiplying frequency of consumption by portion size and nutrient content per gram using the 2011 Dutch food composition table [31]. The Alpha Omega Cohort used a 203-item FFQ, which was an extended version of a previously validated FFQ [29]. Food-consumption data were converted into energy and nutrient intake by means of the 2006 Dutch food composition database [32]. In Predimed-Plus, a validated 143-item semi-quantitative FFQ was used to assess dietary intake [33, 34]. Reported food consumption frequencies were converted to number of intakes per day and multiplied by portion sizes specified in the questionnaire. The Spanish food composition tables were used to derive energy and nutrient intake [35, 36]. In Feel4diabetes, dietary intake was assessed with a 33-item FFQ for food groups and beverages [37]. In contrast to the other studies, nutrients and total energy intakes were not derived from this frequency questionnaire. To calculate intakes in grams per day for the food groups, the reported frequencies were multiplied by the indicated portion size. When the lowest frequency category was “less than one serving”, the average between no intake and one serving was taken. When the lowest category was “One or less than one serving per week” or the highest category was “5 or more”, the given portion size for one serving or for five servings was used, respectively. In Feel4diabetes, beverage consumption was assessed by means of an additional questionnaire by asking for the consumption frequency (glass/week) of soft drinks with and without sugar as well as other beverages (1 glass = 250 ml), i.e. water, coffee, tea, juices and alcoholic drinks. All cohorts included intake measures of SSBs and LNCBs, except for the Alpha Omega Cohort where only SSB consumption was available. For the current work, SSB consumption was defined as soda, sugary drinks or lemonade, and LNCB consumption was defined as items under “diet soda” in the FFQs. For the purpose of this analysis, a standardised serving of 150 mL was calculated in all studies based on the smallest standard packaging for soft drinks.

### Covariates

In all cohorts, age, sex, educational level, smoking status and medical history were assessed with either self- or interview-administered questionnaires. Educational level was categorised into less than secondary school qualification (low), secondary school diploma up to university classes but no Bachelor’s degree (medium), and Bachelor, Master or PhD degree (high). Smoking status was categorised into never, former, or current. Participant history of diseases (type 2 diabetes, cardiovascular diseases (CVD), cancer, hypertension and high cholesterol) was assessed by self-report or medical staff at recruitment and at subsequent visits. In Lifelines and NQplus, physical activity was assessed using the Short Questionnaire to Assess Health (SQUASH) [38] and the Activity Questionnaire for Adults and Adolescents (AQuAA) [39]. Physical activity is, thus, reported as MET-min/week for light, moderate and intense exercise and sedentary behaviour in min/week (i.e. TV watching or sitting time). In the Alpha Omega Cohort, the validated Physical Activity Scale for the Elderly (PASE) was used [40]. Participants were subsequently categorised as sedentary (0 METs), light (0 to ≤ 3 metabolic equivalents [METs]), moderate (> 0 to < 5 days/week of moderate or vigorous activity, > 3 METs) or high (≥ 5 days/week of moderate or vigorous activity). In Predimed-Plus, information on physical activity was collected via the Physical Activity Readiness Questionnaire (PAR-Q), the Rapid Assessment of Physical Activity Questionnaires 1 and 2 (RAPA-1 and RAPA-2) [41], the Nurses’ Health Study sedentary lifestyle Questionnaire [42] and the REGICOR Short Physical Activity Questionnaire [43] and reported in METs-min/week. In Feel4diabetes, the physical activity questionnaires included question on frequency (days/week) of sedentary (TV watching, computer etc.), light (walking), moderate and vigorous activities derived from the International Physical Activity Questionnaire (IPAQ) [26, 44].

### Data assessment and harmonisation

**Fig. 1:**
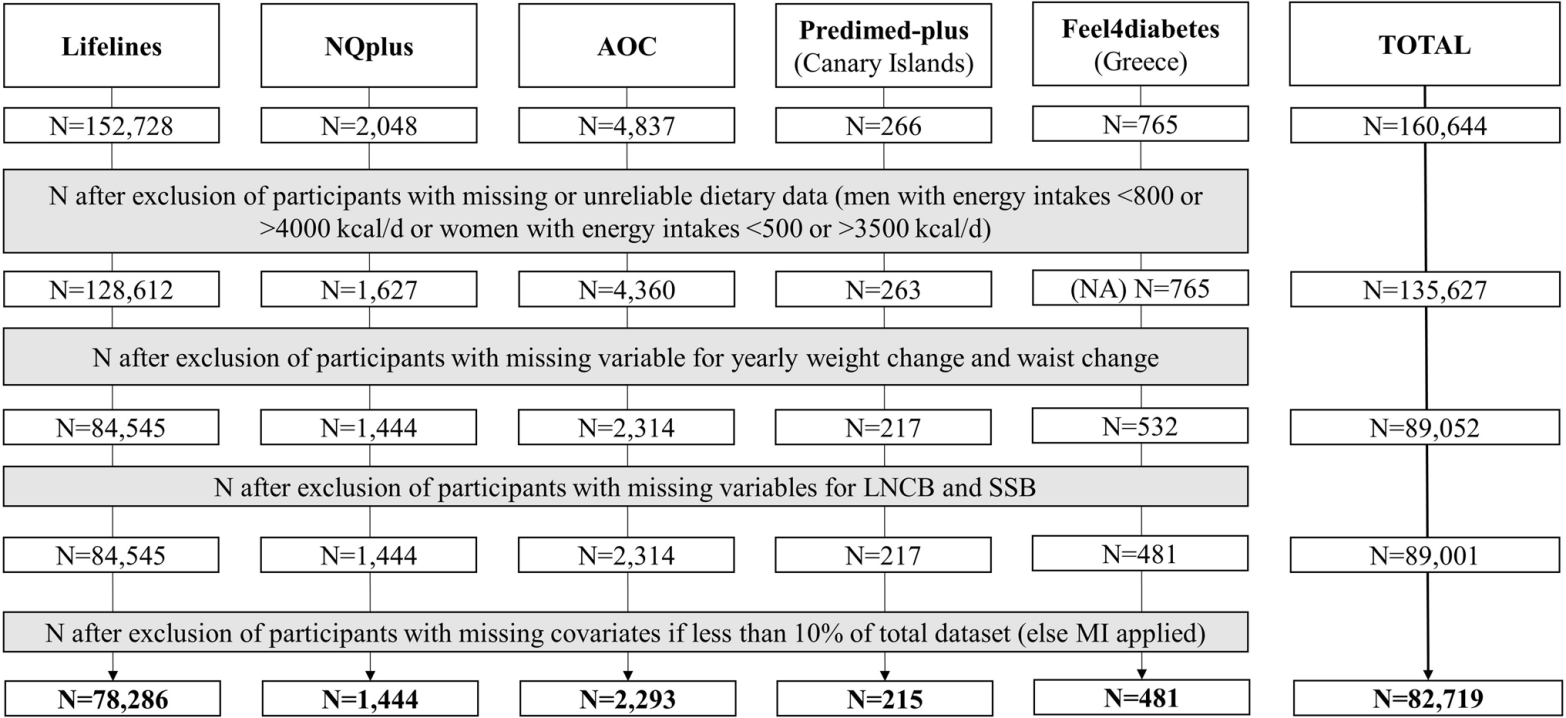
Flow chart of the population studies included in the meta-analyses on sugar-sweetened beverages and low/no-calorie beverages with body weight and waist circumference changes. *AOC* Alpha Omega Cohort, *LNCBs* low/non-calorie beverages, *MI* multiple imputation, *NA* not applicable, *SSBs* sugar-sweetened beverages

Data in the SWEET study have been collected within the framework of independent population studies, with different protocols for data collection and distinct original research foci. Therefore, for the current analysis, harmonised variables were created as far as possible for all parameters of interest (Suppl. Table 2). After exclusion of participants with missing or unreliable dietary data (men with habitual energy intakes < 800 or > 4000 kcal/d or women with habitual energy intakes < 500 or > 3500 kcal/d [45] where available (i.e. all except the Feel4diabetes study) (*n* = 25,017)), exclusion of participants with missing data for outcome and exposure (*n* = 46,626) and exclusion of participants with missing covariates if these participants accounted for less than 10% of total dataset (*n* = 6282) [46, 47], a total of *n* = 82,719 participants were included for the prospective analyses (Fig. 1). In both NQplus and Feel4diabetes, participants with missing covariates accounted for 10–15% of the total dataset; thus, multiple imputation was used to impute the missing values in these two cohorts. Multiple imputation was conducted with the mice package in R using participants characteristics included in the current analysis.

### Statistical analysis

Baseline characteristics of each cohort are presented by mean (SE), median (IQR) or % where appropriate. Differences across categories of SSB and LNCB consumption were assessed using ANOVA or Kruskal–Wallis for continuous variables and Chi-square tests for categorical variables. Previous restricted cubic spline analyses with data of the Lifelines cohort did not show strong evidence for non-linear associations between SSB or LNCB consumption and body weight and waist circumference changes [12], and further exploration in other cohorts did not produce further evidence (data not shown). Thus, multiple linear regression analyses were conducted to assess associations between baseline daily SSB and LNCB consumption, and yearly body weight and waist circumference changes in each cohorts.

To investigate the association with weight-related outcomes when theoretically replacing each serving of SSB with a serving of either LNCB or the replacement of either SSB or LNCB consumption with water, so-called substitution analyses were conducted by means of a leave-one-out model [48]. This model included the sum of all beverages as one variable followed by the beverages defined as replacement, as well as all other confounders as modelled in the analyses. Potential confounders were identified based on a priori knowledge and separate cohort analyses were adjusted for similar available confounders as much as possible. Overall, models were adjusted for sex and age, baseline body weight or baseline waist circumference, and height, education (low, medium and high), physical activity (light, moderate and intense in Metabolic equivalent of task (MET)-min/week or min/week), sedentary behaviour (min/week), alcohol intake (none, low, medium and high), smoking (non-smokers, former or current), dietary variables, namely meat, milk and milk products, vegetables, legumes, grains, fats and oils, potatoes, nuts, fruits, other beverages (tea, coffee, fruit juice and fruit drinks), sugary snacks intakes (g/d) and total energy intake (only for LNCBs). As total energy can be an intermediate in the association between SSB consumption and body weight [49], we present the models unadjusted for total energy. Moreover, as participants of Alpha Omega Cohort, Predimed-Plus and Feel4diabetes were originally randomised across control and intervention groups, associations assessed in these studies were additionally adjusted for intervention group. More details on the adjustments performed in each cohort are presented in Suppl. Table 1. Subsequently, an interaction term with BMI (< 25 kg/m^2^ [normal-weight] and ≥ 25 kg/m^2^ [overweight/obese]) and sex was added to the adjusted model to test for effect modification and stratified analyses were performed. Furthermore, we conducted sensitivity analyses, excluding participants with history of diseases at baseline (type 2 diabetes, CVD, cancer, hypertension and hypercholesterolemia). Both the Predimed-Plus and Alpha Omega Cohort participants met at least one of the conditions for exclusion in the sensitivity analyses and were consequently fully excluded from these analyses. Estimates from all cohorts were pooled using random-effects meta-analysis. All analyses were performed using R 3.6.1 and RStudio 1.0.

## Results

General characteristics of each cohort are presented in Table 1. Most studies included more women (60–68%) than men, except for NQplus (47%) and the Alpha Omega Cohort (21%). Age and education also differed across the five cohorts with mean age ranging from 44 to 69 years old and lower education from 1% (NQplus) to 72% (Predimed-Plus). Predimed-Plus exclusively included participants with overweight and obesity whilst Lifelines and NQplus had the highest percentage of normal-weight participants (45 and 46%, respectively). Mean ± SD yearly changes in body weight and waist circumference ranged from − 0.90 ± 2.48 kg/y and − 0.78 ± 3.00 cm/y in Predimed-Plus to + 0.11 ± 3.08 kg/y and + 0.10 ± 3.56 cm/y in Feel4diabetes.

**Table 1:** Baseline characteristics of the five EU prospective cohort studies used in the meta-analyses on SSB and LNCB consumption, and body weight and waist circumference changes

Characteristics^a^	Lifelines	NQplus	AOC	Predimed-Plus	Feel4diabetes
*N*	78,286	1444	2293	215	481
Demographics					
Women, %	59.6	47.4	21.2	67.9	63.8
Age, years	45.9 ± 12.7	53.4 ± 11.5	68.8 ± 5.4	65.0 ± 4.5	43.5 ± 7.2
Education, %					
Low	3.9	0.7	56.9	71.6	8.3
Intermediate	64.7	44.1	31.1	25.1	55.7
High	31.3	55.2	12.0	3.3	36.0
Anthropometrics					
Baseline weight, kg	79.5 ± 15.0	79.0 ± 14.5	82.3 ± 12.4	84.2 ± 11.7	80.6 ± 18.0
Waist circumference, cm	90.1 ± 12.2	91.4 ± 12.3	101.3 ± 10.0	106.6 ± 10.0	94.3 ± 14.1
Height, cm	174.7 ± 9.3	174.5 ± 8.8	172.2 ± 8.1	161.3 ± 8.3	167.2 ± 9.1
Baseline BMI, kg/m^2^	26.0 ± 4.2	25.9 ± 4.0	27.7 ± 3.7	32.3 ± 3.5	28.7 ± 5.6
BMI categories, %					
Normal weight	45.0	46.1	21.9	0	28.3
Overweight	40.1	40.1	54.7	30.7	37.2
Obese	14.9	13.8	23.4	69.3	34.5
Lifestyle					
Physical activity, MET- value, min/week or %^b,c^					
Intense	0 (630)	420 (1398)	38.9	168 (993)	0 (180)
Moderate	1665 (2142)	810 (1470)	21.2	490 (1399)	60 (240)
Light	0 (0)	0 (0)	34.9	336 (839)	180 (380)
Sedentary, min/week or %^d^	840 (630)	1920 (1500)	5.0	1740 (1230)	1680 (2520)
Smoking^e^					
Never	46.6	51.6	17.0	68.8	43.5
Former	35.0	40.9	67.4	27.0	24.7
Current	18.5	7.5	15.6	4.2	31.8
Alcohol intake, %^f^					
None	2.5	4.4	4.3	43.3	41.9
Low	71.4	55.2	52.3	44.7	20.3
Medium	19.2	20.4	19.7	12.1	18.4
High	7.0	20.1	23.7	0.0	19.3
History of diseases, %					
Type 2 diabetes	2.4	3.2	14.5	31.2	19.1
CVD	2.3	2.8	100	14.9	NA
Hypertension	22.4	24.7	49.5	90.2	25.8
Hypercholesterolemia	14.1	19.3	1.3	87.4	6.7
Cancer	4.8	5.1	9.9	4.7	NA
Outcomes					
Body weight change, kg/y	0.02 ± 1.58	− 0.20 ± 2.77	− 0.04 ± 1.36	− 0.90 ± 2.48	0.11 ± 3.08
Waist circumference change, cm/y	0.01 ± 2.04	0.18 ± 3.80	0.06 ± 1.80	− 0.78 ± 3.00	0.10 ± 3.56

*AOC* Alpha Omega Cohort, *BMI* body mass index, *CVD* cardiovascular diseases, *MET* metabolic equivalent of task

^a^Mean ± SD or %

^b^METs-min per week in Lifelines, NQplus and Predimed-Plus; min/week in the Feel4diabetes study and categorical in the Alpha Omega Cohort: light (> 0–3 METs), moderate (> 0 to < 5 days/week of moderate or vigorous activity, > 3 METs) or high (≥ 5 days/week of moderate or vigorous activity)

^c^86 participants with missing values for all physical activity measures in NQplus, 74 participants with missing values for sedentary activity and 10 participants with missing values for moderate physical activity in the Feel4diabetes study

^d^Min/week or % of participants with no activity in the Alpha Omega Cohort

^e^71 participants with missing values for smoking in NQplus

^f^Alcohol intake is expressed as g/d of pure ethanol for all cohort and categorised into none (0 g/d), low (> 0–10 g/d), medium (> 10–20 g/d) or high (> 20 g/d), except in the Feel4diabetes study where categorisation was: no alcoholic beverages (0 g/d), > 0-50 g, > 50-95 g, and > 95 g

The proportion of SSB consumers ranged from 28% in Predimed-Plus to 62% in Lifelines (Table 2). The median (IQR) SSB consumption across cohorts ranged from: 0.1 (0.6) servings/d in Lifelines, 0.0 (0.1) in NQplus, 0.1 (0.4) in the Alpha Omega Cohort, 0.0 (0.1) in Predimed-Plus to 0.0 (0.2) servings/d in Feel4diabetes. The proportion of LNCB consumers ranged from 26% in Predimed-Plus to 57% in Lifelines (Table 2). The median (IQR) LNCB consumption across cohorts ranged from: 0.1 (0.6) servings/d in Lifelines, 0.0 (0.1) in NQplus, 0.0 (0.1) in Predimed-Plus to 0.0 (0.2) servings/d in Feel4diabetes. Compared to non-consumers, SSB consumers were more likely to be men, and younger. In the Lifelines cohort, BMI was slightly lower amongst SSB consumers, but no differences were observed in other cohorts. Compared to non-consumers, LNCB consumers were slightly younger than non-consumers in most cohorts. In Lifelines, those consuming LNCBs were more likely to be women, whilst this was not observed in other cohorts. BMI was slightly higher amongst LNCB consumers in all cohorts, except Predimed-Plus. In all cohorts, a higher SSB consumption was associated with a higher total energy intake. In contrast, a higher LNCB consumption was not associated with a higher total energy intake, except in NQplus. More details on the baseline characteristics per category of SSB and LNCB consumption are presented in Suppl. Tables 3 and 4.

**Table 2:** Baseline dietary intakes of the five EU prospective cohort studies used in the meta-analyses on SSB and LNCB consumption, and body weight and waist circumference changes

Dietary variable^a^	Lifelines	NQplus	AOC	Predimed-Plus	Feel4diabetes
*N*	78,286	1444	2293	215	481
Total energy, kcal/d	1977 (740)	1987 (736)	1863 (664)	2089 (749)	NA
Fruits, g/d	110 (178)	209 (155)	115 (212)	371 (215)	71 (116)
Vegetables, g/d	108 (74)	146 (107)	71 (42)	263 (171)	196 (250)
Dairy, g/d	269 (230)	281 (246)	232 (216)	457 (374)	120 (240)
Meat, g/d	76 (43)	68 (55)	78 (64)	102 (62)	94 (70)
Grains, g/d	180 (94)	187 (115)	168 (83)	105 (84)	120 (250)
Potatoes, g/d	88 (58)	66 (58)	99 (72)	50 (64)	NA
Fats and oils, g/d	22 (20)	25 (25)	33 (28)	20 (14)	NA
Sugary snacks, g/d	63 (55)	42 (42)	62 (66)	47 (56)	20 (9)
Legumes, g/d	12 (30)	38 (59)	21 (25)	50 (45)	57 (71)
Nuts, g/d	8 (14)	12 (16)	3 (6)	13 (25)	6 (4)
Coffee, ml/d	465 (349)	406 (464)	375 (188)	50 (75)	286 (321)
Tea, ml/d	232 (303)	174 (339)	150 (396)	0 (3)	0 (36)
Juice, ml/d	27 (92)	21 (90.6)	62 (146)	57 (171)	36 (107)
SSBs, servings/d	0.1 (0.6)	0.0 (0.1)	0.1 (0.4)	0.0 (0.1)	0.0 (0.2)
Range	0 to 11.0	0 to 10.9	0 to 9.0	0 to 1.3	0 to 3.6
SSB categories, %					
Non-consumers	37.9	56.6	47.3	72.6	68.0
≤ 2servings/week	25.8	30.1	20.2	20.5	11.8
> 2servings/week	36.3	13.4	32.5	7.0	20.0
LNCBs, servings/d	0.1 (0.6)	0.0 (0.1)	NA	0.0 (0.1)	0.0 (0.2)
Range	0 to 11.1	0 to 5.6		0 to 3.3	0 to 10.0
LNCB categories, %					
Non-consumers	43.4	67.4	NA	74.4	65.3
≤ 2servings/week	21.2	19.1		18.6	12.9
> 2servings/week	35.4	13.5		7.0	21.8
Water, servings/d ^b^	1.9 (11.1)	0.0 (0.1)	NA	6.0 (2.7)	8.3 (6.9)

*AOC* Alpha Omega Cohort, *LNCBs* low/no-calorie beverages, *NA* not available, *SSBs* sugar-sweetened beverages

^a^Median (IQR) or %

^b^Number of participants with data on water consumption were *N* = 22,859; *N* = 1444; *N* = 156 and *N* = 479 for Lifelines, NQplus, Predimed-Plus and Feel4diabetes, respectively

**Fig. 2:**
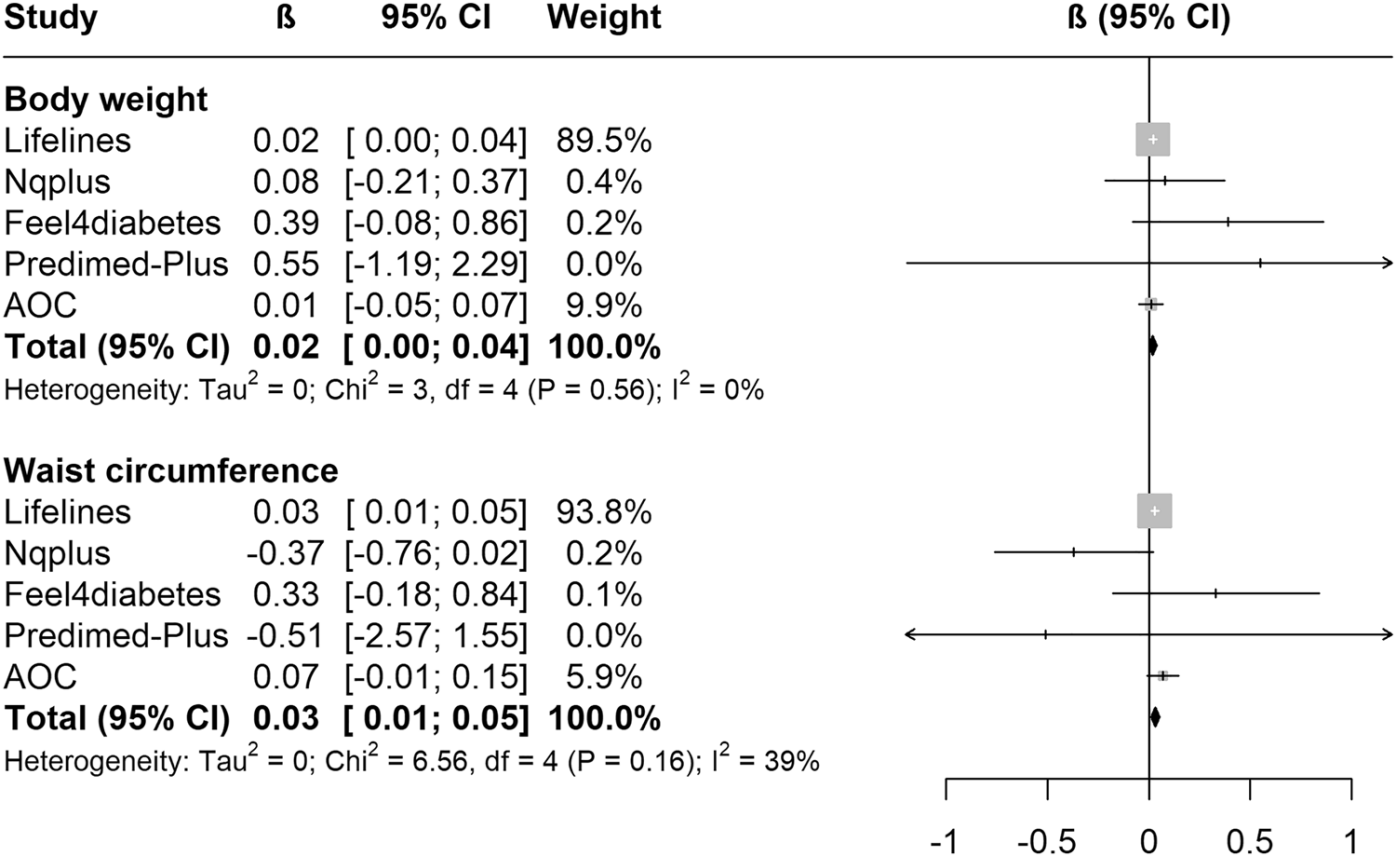
Meta-analyses of sugar-sweetened beverages (SSBs) with yearly body weight (kg) and waist circumference (cm) changes in the five EU prospective cohort studies. The black square is the pooled estimate of the random-effects model and represents the yearly body weight or waist circumference change for one serving of SSB. “Weight” refers to the weight of each study in the random-effects meta-analysis. Models were adjusted for age, sex, group, baseline weight (or waist circumference) and height, education, physical activity, sedentary behaviour, alcohol intake, smoking and all dietary data. *AOC* Alpha Omega Cohort

**Fig. 3:**
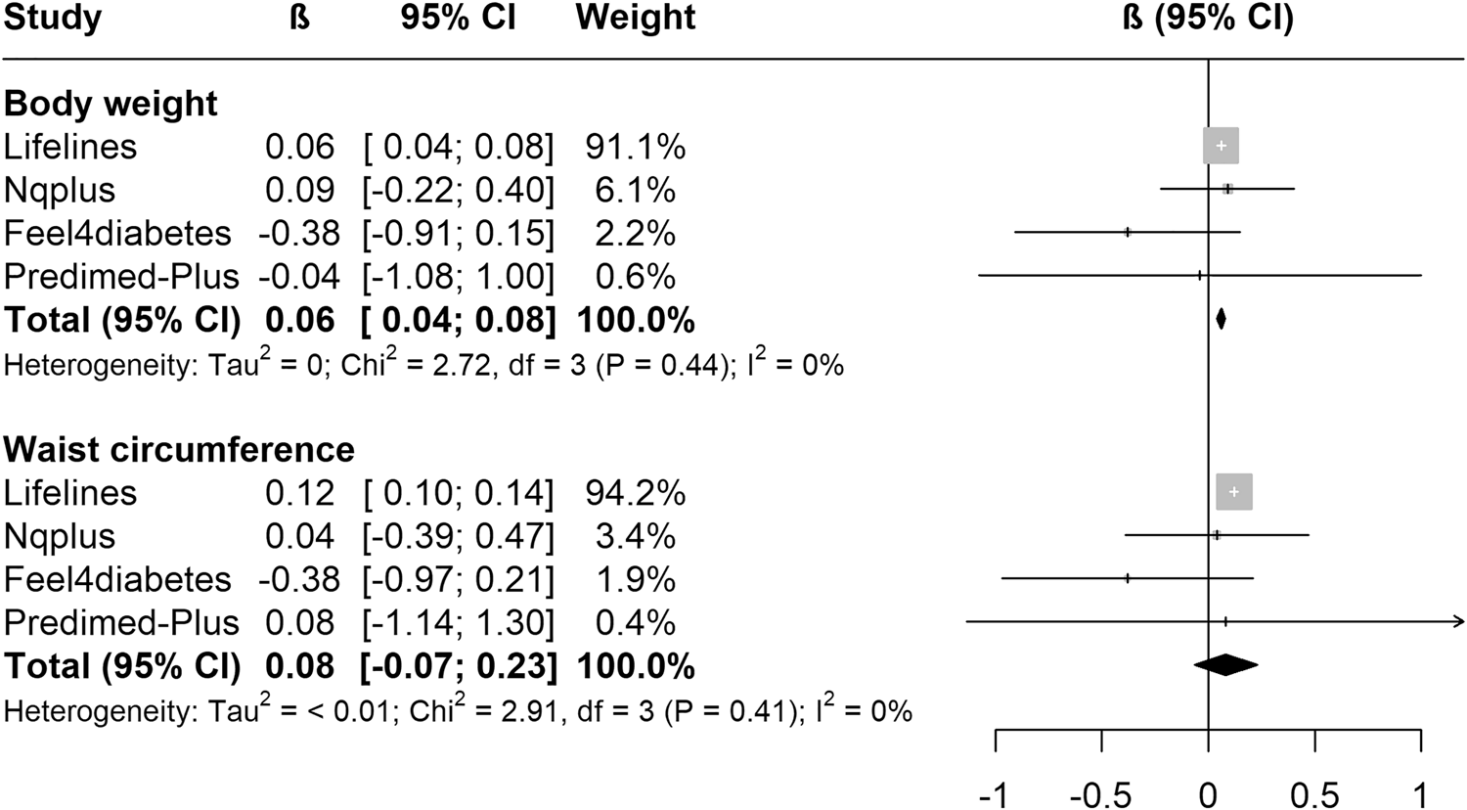
Meta-analyses of low/no-calorie beverages (LNCBs) with yearly body weight (kg) and waist circumference (cm) changes in the five EU prospective cohort studies. The black square is the pooled estimate of the random-effects model and represents the yearly body weight or waist circumference change for one serving of LNCB. “Weight” refers to the weight of each study in the random-effects meta-analysis. Models were adjusted for age, sex, group, baseline weight (or waist circumference) and height, education, physical activity, sedentary behaviour, alcohol intake, smoking; all dietary data and total energy intake

Random-effects meta-analysis pooling five cohorts using fully adjusted models indicated a weak positive association between an increase by one serving SSB/day and body weight change (+ 0.02 kg/y, 95%CI 0.00; 0.04; *I*^2^ = 0%) and waist circumference change (+ 0.03 cm/y, 95%CI 0.01; 0.05; *I*^2^ = 39%, *P*_heterogeneity_ = 0.16) (Fig. 2). Each increase in one serving/day LNCB (*N* = 4 cohorts) was also positively associated with higher body weight change (+ 0.06 kg/y, 95%CI 0.04; 0.08, *I*^2^ = 0%), but not with waist circumference change (+ 0.08 cm/y, 95%CI − 0.07; 0.23, *I*^2^ = 0%) (Fig. 3).

**Fig. 4:**
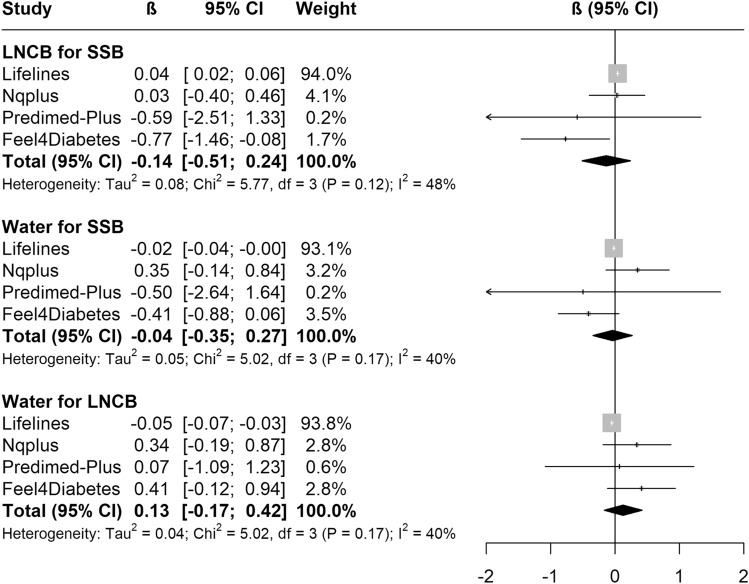
Meta-analyses of the association between theoretical substitution of beverages and yearly body weight change (kg/y) in the five EU prospective cohort studies. The black square is the pooled estimate of the random-effects model and represents the yearly body weight change for each theoretical substitution. “Weight” refers to the weight of each study in the random-effects meta-analysis. Models were adjusted for age, sex, group, baseline weight or waist circumference and height, education, physical activity, sedentary behaviour, alcohol intake, smoking; all dietary data and total energy intake (only models of water as substitute for LNCBs). *LNCBs* Low/no-calorie beverages, *SSBs* sugar-sweetened beverages

**Fig. 5:**
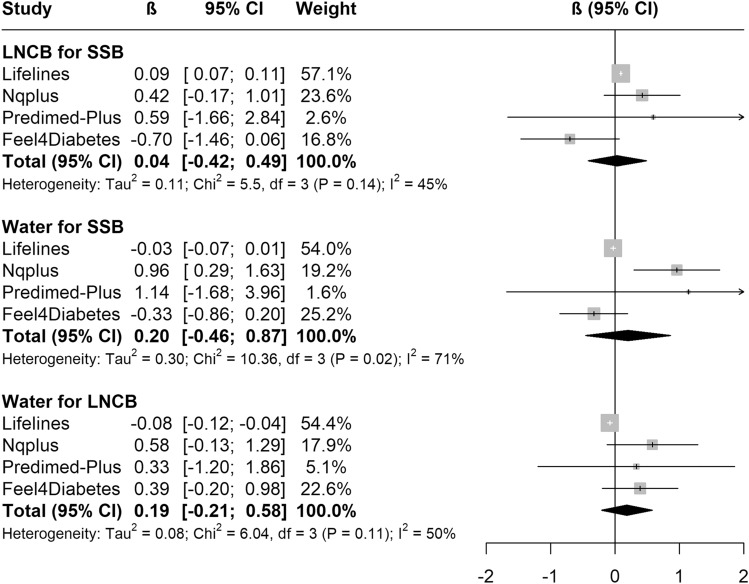
Meta-analyses of the association between theoretical substitution of beverages and yearly waist circumference change (cm/y) in the five EU prospective cohort studies. The black square is the pooled estimate of the random-effects model and represents the yearly waist circumference change for each theoretical substitution. “Weight” refers to the weight of each study in the random-effects meta-analysis. Models were adjusted for age, sex, group, baseline weight or waist circumference and height, education, physical activity, sedentary behaviour, alcohol intake, smoking; all dietary data and total energy intake (only models of water as substitute for LNCBs). *LNCBs* low/no-calorie beverages, *SSBs* sugar-sweetened beverages

The results for theoretical replacement of beverages in the four cohorts with available data are shown in Figs. 4 and 5. After adjusting for baseline anthropometrics, lifestyle and health variables, none of the theoretical replacements were associated with body weight or waist circumference change. Details on all models presented above are available in Suppl. Tables 5 and 6.

Pooled associations stratified by BMI category indicated that the association between an increase of one serving/day SSB and body weight change was most pronounced amongst normal-weight participants compared to participants with overweight or obesity (+ 0.06 kg/y, 95%CI − 0.01; 0.12; 36,506 participants versus − 0.00 kg/y, 95%CI − 0.02; 0.02; 46,213 participants). No other relevant difference in the main models was observed. However, theoretically replacing one serving/day SSB with an equal serving LNCB resulted in an adverse association with waist circumference change in normal weight participants (+ 0.05 cm/y, 95%CI 0.01; 0.09, *I*^2^ = 0%) and women (+ 0.10 cm/y, 95%CI 0.06; 0.14, *I*^2^ = 0%). Women also showed beneficial associations with waist circumference change when theoretically replacing one serving/day SSB with water (− 0.06 cm/y, 95%CI − 0.12; 0.00, *I*^2^ = 10%, *P*_heterogeneity_ = 0.34) and a serving/day LNCB with water (− 0.10 cm/y, 95%CI − 0.14; − 0.06, *I*^2^ = 0%). No other relevant difference upon stratification was observed. Details on stratified results are available in Suppl. Tables 7 and 8.

Further sensitivity analyses excluding participants with a history of diseases (*N* = 54,694 participants; 3 cohorts), affected the associations in inconsistent directions between SSBs, LNCBs and body weight outcomes. The adverse association between SSB consumption and waist circumference change disappeared whilst the association between LNCB consumption and waist circumference change became statistically significant (+ 0.12 cm/y, 95%CI 0.10; 0.14). Theoretically substituting water for LNCBs became associated with lower body weight and waist circumference changes (− 0.05 kg/y, 95%CI − 0.09; − 0.01 and − 0.08 cm/y, 95%CI − 0.12; − 0.04); however, other theoretical substitutions were not affected (Additional details in Suppl. Table 9).

## Discussion

In this meta-analysis of five European prospective cohort studies, SSB consumption was weakly and positively associated with body weight and waist circumference change. LNCB consumption was positively associated with body weight change but was not significantly associated with waist circumference changes. Overall, no association was observed in the theoretical replacement of SSBs with LNCBs, neither in the replacement of SSBs or LNCBs with water.

The role of LNCS on weight change is still a large debate. Most clinical studies report a beneficial effect of using LNCS on weight management in the short term [1, 2, 5, 6, 8]. In contrast, previous meta-analyses either report weak associations with body weight [5] or BMI change [2, 5, 6], or no association in the long term [1]. Only one other meta-analysis included studies that considered waist circumference change as outcome with three cohorts and also did not find any evidence of an overall association with waist circumference [6]. Despite the harmonisation of the different datasets and standardised covariate adjustment in this meta-analysis, our findings are in line with previous meta-analyses, that is, results were not consistent across studies. This may be potentially explained by the populations being different at baseline. For example, Feel4diabetes included participants at higher risk of developing type 2 diabetes and Predimed-Plus was exclusively composed of participants with overweight and obesity at baseline, whereas NQplus was healthier and more highly educated compared to other studies.

In this study, we also reported that participants with overweight or obesity consumed more LNCBs at baseline. These participants may consume LNCBs in an attempt to maintain or lose weight whilst reaching different end results. This may explain the different outcome from one population to the other within prospective studies and in comparison to clinical trials. The results of our sensitivity analyses, however, showed an adverse association when excluding participants with a history of diseases, who might be more prone to adapt their dietary intakes. Whilst these secondary analyses do not completely exclude reverse causality, the observed adverse associations may be explained by other non-biological reasons such as a compensation with other unhealthy foods when low/no-calorie sweeteners are consumed [50]. Biological reasons have also been reported, such as an activation of the sweet taste reception and subsequent insulin secretion leading to weight gain and/or disruption of the gut microbiota [51, 52]. However, the evidence for these mechanisms is still inconsistent or limited in clinical trials when compared to water or unsweetened products [1, 53–55]. In trials, LNCS are often consumed instead of—or compared to—added sugars, whilst this might not be as simple in reality where participants simultaneously consume both, which might also explain the overall differences between experimental and observational studies [6, 56]. The potential effect of an interaction between sugar and LNCS cannot be overlooked in the current work. Although the statistical models of SSB and LNCB consumption with weight outcomes adjusted for one another, there could still be sugar or other sweeteners from other sources not accounted for in the FFQs. Nonetheless, SSB and LNCB consumption was not correlated in most cohorts (data not shown).

Substituting one beverage for the other, including water, also showed no association in this study. Only a few individual cohort studies have attempted to study these substitutions [9–11]. Pan and colleagues reported ~ 0.45 kg less weight gain for a 4-y period when one daily serving LNCB is a substitute for an equal serving SSB as well as a ~ − 0.5 kg reduction in weight gain per 4-year period when water is a substitute for SSBs, amongst > 120,000 participants. Over a similar period, Fresán and colleagues reported a − 205 g (− 187 g (95% CI − 425 to 16) when water is a substitute for SSBs, in > 15,000 adults. In contrast, other studies did not find an association for water as substitute [11] and LNCBs as substitute [10, 11] on weight and waist change, which is in line with our results. The lack of association could be owed to the explanations mentioned in previous paragraphs (i.e. differences in populations and/or reverse causality). In addition, unhealthy foods, such as SSBs, have also been shown to be under-reported more than foods that are perceived as healthy [57]. Under-reporting could explain the lack of association in substitution of LNCBs or water for SSBs, since SSB consumption might not be accurately estimated. By the same token, it could explain the overall weak associations between SSBs and weight or waist change. This may be further confirmed by the stronger association observed between SSBs and weight change in participants with normal weight compared to participants with overweight or obesity that are known to under-report more than participants with healthy weight [57].

This study has several strengths. Previous meta-analyses used summary data where associations of individual studies were adjusted for a wide range of confounders that differed per study [1, 2]. This is the first meta-analysis on sweet beverages and weight measures using harmonised individual participant data, also including theoretical substitution models. Cohorts included in this work also measured body weight and waist circumference rather than using self-reported measures. Additionally, where most of prospective studies included in previous analyses were conducted in the US, we explored prospective associations between sweetened beverages and body weight and waist circumference changes in European adults. However, out of the five studies, three were from the Netherlands. Thus, the overall results might not be representative of the European population. Although populations included in our meta-analyses differed at baseline, we also consider this a strength in terms of generalisation of our findings. However, it is important to note that the harmonisation process was not always optimal: some sources of heterogeneity could not be addressed in the harmonisation due to differences in study set-up and assessment methods, which may compromise comparability. Additional limitations of our study include the lower consumption levels of SSBs and LNCBs with median intakes lower than 150 mL per day compared to RCTs in which dosage ranged between 250 and 2000 mL per day for LNCBs and 250 and 1750 mL per day for SSBs [8]. The lower consumption of LNCBs and SSBs in this meta-analysis could explain the relatively weak or absent associations in our study. Moreover, our study included the use of general FFQs as with most other large-scale prospective studies. In addition to being self-reported and, as such, being prone to recall bias and other measurement errors, the FFQs used in these cohorts were not specifically designed for the purpose of investigating LNCS consumption. Specifically, the FFQs did not account for foods that contain LNCS and did not allow differentiation in the type of sweeteners consumed [58]. Future research on LNCS in both observational studies and RCTs should focus on specific sweeteners and blends compared to general LNCB consumption, to consider the distinct chemical and metabolism properties of different sweeteners that could play a role in the interpretation of the findings [59]. Furthermore, the analyses were conducted with baseline data of participants, representing habitual consumption. Repeated dietary assessment could have reflected dietary changes during the course of follow-up and might further minimise the effect of reverse causality. Nonetheless, this work was still able to investigate associations between habitual consumption and weight outcomes. Finally, as with all observational studies, and despite the adjustment for a large set of confounders, residual confounding cannot be entirely ruled out.

In conclusion, this meta-analysis of five European studies showed overall positive associations of SSB consumption with long-term weight and waist circumference changes whilst LNCB consumption was positively associated with weight change only. Theoretical replacement of SSBs with water or LNCBs and replacement of LNCBs with water were not associated with any outcomes. Thus, the potential benefit of LNCBs as an alternative to SSBs remains unclear. Future prospective research with more specific and accurate dietary assessment of SSBs and LNCBs might address the current inconsistencies in this area.

## Supplementary Information

Below is the link to the electronic supplementary material.

Supplementary file1 (DOCX 139 KB)

## Data Availability

Data described in the manuscript, code book, and analytic code will be made available upon request pending application and approval.
