# Supplementary material for: Sugar and low/no-calorie-sweetened beverage consumption and associations with body weight and waist circumference changes in five European cohort studies: the SWEET project
Source: Eur J Nutr. 2023 Jul 5;62(7):2905–18. doi: 10.1007/s00394-023-03192-y (PMC10468933; doi:10.1007/s00394-023-03192-y)
Supplement: Supplementary file 1 — Supplementary file1 (DOCX 139 KB) [file 394_2023_3192_MOESM1_ESM.docx]

**SUPPLEMENTAL MATERIAL: Sugar and low/no-calorie-sweetened beverage consumption and associations with body weight and waist circumference changes in five European cohort studies: The SWEET project**

**Supplemental Tables**

[Supplemental Table 1. Characteristics of the five EU prospective cohort studies included in the meta-analyses of sugar-sweetened beverages (SSBs) and low/non-calorie beverages (LNCBs) with yearly body weight and waist circumference change. 2](#_Toc138252788)

[Supplementary Table 2. Harmonized variables of the five EU prospective cohort studies included in the meta-analyses of sugar-sweetened beverages (SSBs) and low/non-calorie beverages (LNCBs) with yearly body weight and waist circumference change. 4](#_Toc138252789)

[Supplemental Table 3. Baseline characteristics of the EU prospective cohort studies by category of sugar-sweetened beverages. 10](#_Toc138252790)

[Supplemental Table 4. Baseline characteristics of the EU prospective cohort studies by category of low/no-calorie beverages. 12](#_Toc138252791)

[Supplemental Table 5. Meta-analyses of sugar-sweetened beverages (SSBs) and low/non-calorie beverages (LNCBs) with yearly body weight and waist circumference change in the EU prospective cohort studies. 14](#_Toc138252792)

[Supplemental Table 6. Meta-analyses of the associations between the theoretical substitution of one serving beverage for another and yearly body weight and waist circumference change in the EU prospective cohort studies. 15](#_Toc138252793)

[Supplemental Table 7. Meta-analyses of sugar-sweetened beverages (SSBs) and low/non-calorie beverages (LNCBs) with yearly body weight and waist circumference change stratified by BMI and sex in the EU prospective cohort studies. 16](#_Toc138252794)

[Supplemental Table 8. Meta-analyses of the associations between the theoretical substitution of one serving beverage for another and yearly body weight and waist circumference change stratified by BMI and sex in the EU prospective cohort studies. 17](#_Toc138252795)

[Supplemental Table 9. Sensitivity analyses of sugar-sweetened beverages (SSB) and low/no-calorie beverages (LNCB) with body weight and waist circumference change after excluding participants with history of diseases (type 2 diabetes, cardiovascular diseases, cancer, hypertension and/or hypercholesterolemia) ^a^. 19](#_Toc138252796)

Supplemental Table 1. Characteristics of the five EU prospective cohort studies included in the meta-analyses of sugar-sweetened beverages (SSBs) and low/non-calorie beverages (LNCBs) with yearly body weight and waist circumference change.

| **Population study** | **N included** | **Main characteristics** | **Baseline** | **Follow-up** | **Follow-up period** | **Dietary assessment** | **Definitions of exposure** | **Fully adjusted models** |
| --- | --- | --- | --- | --- | --- | --- | --- | --- |
| Lifelines Cohort Study (The Netherlands) | 78,286 | 0-70y M+F | 2006  -2013 | 2011  -2018 | 4y (median) | FFQ | SSB = soft drinks and lemonade (soda, lemonade, sport drinks and energy drinks) LNCB = Light soft drink, light lemonade | age, sex, baseline weight or waist circumference, height, education, physical activity, sedentary behaviour, alcohol intake (ethanol), smoking; all dietary intake data (intakes of meat and processed meat, dairy, vegetables, legumes, fruits, juices, sugary snacks, potatoes, coffee, tea, grains and cereals, fats and oils, nuts and mutual adjustments for LNCB or SSB), and total energy intake |
| NQPlus (The Netherlands) | 1,444 | 20-70y M+F | 2011  -2013 | 2013  -2015 | 2y | FFQ | SSB = soft drinks and lemonade (soda, lemonade, sport drinks and energy drinks) LNCB = Light soft drink, light lemonade | age, sex, baseline weight or waist circumference, height, education, physical activity, sedentary behaviour, alcohol intake (ethanol), smoking; all dietary intake data (intakes of meat and processed meat, dairy, vegetables, legumes, fruits, juices, sugary snacks, potatoes, coffee, tea, grains and cereals, fats and oils, nuts and mutual adjustments for LNCB or SSB), and total energy intake |
| Alpha Omega Cohort (The Netherlands) | 2,293 | Diagnosed myocardial infraction 60-80y M+F Initially a n-3 fatty acids supplementation intervention | 2002  -2006 | 2012 | 3.3y (40 months) | FFQ | SSB = soft drinks and lemonade (coke with caffeine, other soda, ice tea) | age, sex, group, baseline weight or waist circumference, height, education, physical activity categories, alcohol intake (ethanol), smoking; all dietary intake data (intakes of meat and processed meat, dairy, vegetables, legumes, fruits, juices, sugary snacks, potatoes, coffee, tea, grains and cereals, fats and oils, nuts and mutual adjustments for LNCB or SSB), and total energy |
| Predimed-Plus (Spain) | 215 | Overweight and obese 55-75y M+F Initially a lifestyle intervention | 2013  -2016 | 2015  -2018 | 2y | FFQ | SSB = sugar-sweetened carbonated beverages/soft drinks: cola drinks, lemonades, tonic, etc. • LNCB = low calorie carbonated beverages/light soft drinks | age, sex, group, baseline weight or waist circumference, height, education, physical activity, sedentary behaviour, alcohol intake (ethanol), smoking; all dietary intake data (intakes of meat and processed meat, dairy, vegetables, legumes, fruits, juices, sugary snacks, potatoes, coffee, tea, grains and cereals, fats and oils, nuts and mutual adjustments for LNCB or SSB), and total energy intake |
| Feel4Diabetes (Greece) | 481 | High risk of type 2 diabetes  25-77y M+F Initially a lifestyle intervention | 2016 | 2019 | 2y | FFQ | • SSB = soft drinks with sugar  • LNCB = soft drink without sugar, e.g. coca cola light | age, sex, group, baseline weight or waist circumference, height, education, physical activity, sedentary behaviour, alcohol intake (alcoholic beverages), smoking; all dietary intake data (intakes of meat and processed meat, dairy, vegetables, legumes, fruits, juices, sugary snacks, coffee, tea, grains and cereals, nuts and mutual adjustments for LNCB or SSB). |
| FFQ: Food Frequency Questionnaire; LNCB: Low/no-calorie beverage; SSB: Sugar-sweetened beverage; M: Male; F: Female; Y: Years. | | | | | | | | |

Supplementary Table 2. Harmonized variables of the five EU prospective cohort studies included in the meta-analyses of sugar-sweetened beverages (SSBs) and low/non-calorie beverages (LNCBs) with yearly body weight and waist circumference change.

| Variable/label | Categories/units | Lifelines | NQplus | Predimed-Plus | Feel4diabetes | AOC |
| --- | --- | --- | --- | --- | --- | --- |
| Sex | Male/female |  |  |  |  |  |
| Age | years |  |  |  |  |  |
| Education | low | no education or primary education | no education or primary education | primary school, no reading and writing | 9 years or less | primary or lower secondary education |
|  | medium | lower or preparatory vocational education, lower general secondary education, intermediate vocational education or apprenticeship, or higher general secondary education or pre-university secondary education | lower or preparatory vocational education, lower general secondary education, intermediate vocational education or apprenticeship, or higher general secondary education or pre-university secondary education | technical or trade school, secondary school | 10-14 years | higher secondary or lower tertiary education |
|  | high | higher vocational education, or university | higher vocational education, or university | higher education | >15 years | higher tertiary education |
| Body weight | kg |  |  |  |  |  |
| Waist circumference | cm |  |  |  |  |  |
| Height | cm |  |  |  |  |  |
| Physical activity | intense | all leisure time and commuting activities performed with intensity = 3. if intensity is missing, activities are categorized based on MET-values. MET-value ≥6 is intense (=all sports) | all leisure time and commuting activities performed with intensity = 3. if intensity is missing, activities are categorized based on MET-values. MET-value ≥6 is intense (=cycling, ball sports, athletics, martial arts, dancing or aerobics, skating, swimming, and bicycling) | all leisure time and commuting activities performed with intensity = 3. if intensity is missing, activities are categorized based on MET-values. MET-value ≥6 is intense (=cycling, ball sports, athletics, martial arts, dancing or aerobics, skating, swimming, and bicycling) | time (min/week) used to do all activity considered as vigorous activities. if hour missing (assumed as 1 hour/day) | intense (%) = ≥5 d/w moderate or vigorous active (>3MET) |
|  | moderate | all leisure time and commuting activities performed with intensity = 2. if intensity is missing, activities are categorized based on MET-values. MET-value ≥3-<6 is moderate (=DIY, walking, gardening, horseback riding, and other sports (median value)) | all leisure time and commuting activities performed with intensity = 2. if intensity is missing, activities are categorized based on MET-values. MET-value ≥3-<6 is moderate (=DIY, walking, gardening, horseback riding, and other sports (median value)) | all leisure time and commuting activities performed with intensity = 2. if intensity is missing, activities are categorized based on MET-values. MET-value ≥3-<6 is moderate (=DIY, walking, gardening, horseback riding, and other sports (median value)) | time (min/week) used to do all activity considered as moderate activities. if hour missing (assumed as 1 hour/day) | moderate (%) = >0-5 d/w moderate or vigorous active (>3MET) |
|  | light | all leisure time and commuting activities performed with intensity = 1. If intensity is missing, activities are categorized based on MET-values. MET-value <3 is light (=no activities) | all leisure time and commuting activities performed with intensity = 1. If intensity is missing, activities are categorized based on MET-values. MET-value <3 is light (=no activities) | all leisure time and commuting activities performed with intensity = 1. If intensity is missing, activities are categorized based on MET-values. MET-value <3 is light (=no activities) | time (min/week) walking | light (%) = only light active (<3MET) |
|  | sedentary | television watching (min/week) | total sedentary activities including television watching, computer, reading, and sitting (min/week) | sitting (min/week) | sitting (min/week) | sedentary (%) = no activity |
| Smoking | never | not currently smoking and never smoked for a full year | not currently smoking and never smoked for a full year | never smoked | never smoked | never |
|  | former | not currently smoking, (at least one year) | not currently smoking, (at least one year) | ex-smoker | former smoker | former |
|  | current | currently smoking cigarettes, cigars or pipes a day < 10 and currently smoking cigarettes, cigars or pipes a day ≥ 10 | currently smoking cigarettes, cigars or pipes a day < 10 and currently smoking cigarettes, cigars or pipes a day ≥ 10 | currently smoking cigarettes, cigars or pipes a day < 10 and currently smoking cigarettes, cigars or pipes a day ≥ 10 | current smoker <10, current smoker ≥ 10 | current |
| Alcohol intake | g/d | ethanol | ethanol | ethanol | Wine, beer and spirits | ethanol |
| Hypertension history | Yes/ No | have you ever had hypertension? | have you ever had hypertension? | have you ever been informed by healthcare personnel that you had high blood pressure? |  | have you ever been diagnosed with high blood pressure (hypertension)? |
| Hypercholesterolemia history | Yes/ No | have you ever been diagnosed with high cholesterol? | have you ever been diagnosed with high cholesterol? | have you ever been informed by healthcare personnel that you had high cholesterol levels? |  | na |
| CVD history | Yes/ No | have you ever had a heart attack? **or** have you ever had a stroke? | have you ever had a heart attack? **or** have you ever had a stroke? | have you ever been informed by healthcare personnel that you had a stroke or cerebrovascular accident? |  | all post-mi |
| Diabetes history | Yes/ No | do you have diabetes mellitus? | do you have diabetes mellitus? | have you ever been informed by healthcare personnel that you had diabetes? |  | have you ever been diagnosed you with diabetes (diabetes mellitus)? |
| Cancer history | Yes/No | do you have cancer or have you had cancer? | do you have cancer or have you had cancer? | diagnosis: cancer | na | have you ever been diagnosed with cancer? |
| Total energy | kcal/d |  |  |  | na |  |
| Fruit | g/d | fresh fruit | apples, bananas, pears, oranges, strawberry, grapes, other fruit | orange, grapefruit or tangerines, banana, apple or pear, strawberries, cherries, plums, peach, apricot, nectarine, watermelon, melon, kiwi, grapes, olives | fruits and berries (fresh or frozen or dried fruit or berries) | citrus fruits, apples, pears, bananas, strawberries, blueberries, cherries, grapes, peaches, nectarines, plums, apricot, kiwi, dried fruits and other fruits |
| Vegetables | g/d | boiled vegetables without butter, stir-fried vegetables, boiled vegetables with butter | cauliflower, broccoli, spinach, leak, french beans, carrot, lettuce, tomato, cabbage, raw carrot, other raw vegetables, other vegetables | chard, spinach, cabbage, cauliflower, broccoli, lettuce, endive, escarole, tomato raw, carrot squash, aubergine, courgette, cucumber, peppers, asparagus, gazpacho andaluz, onion, garlic, wild mushrooms, saffron milkcaps, mushrooms, other vegetables | vegetables (tomato, broccoli or leafy vegetables) | endive, spinach and purslane, sprouts, cauliflower, broccoli, other cabbage, carrots, leek, chicory, kohlrabi, beets, mushrooms, bell pepper, onion, other kin of vegetables, tomatoes, lettuce and crudites |
| Grains and cereals | g/d | dutch rusks/knackebrod/crackers, croissants/other breads, bread, muesli/cruesli/breakfast cereals, pasta, rice | croissants, dutch spiced honey cake, muesli/cruesli, cornflakes/smacks/etc, fibre rich breakfast cereals, oatmeal, dutch rusks/crackers, wholemeal dutch rusks/knackebrod, high-fibre/wholemeal knackbrod, rhy bread, white rolls/currant buns, wholemeal breads/muesli buns, multigrain breads, whitebread, wheat bread, wholemeal bread, multigrain bread, currant/raisin bread, white pasta, wholemeal pasta, white rice, brown rice | bread, white, loaf, baguette, bread, brown or wholegrain, croissants, whole grain crackers, breakfast cereals, wholegrain cereals: muesli, oat flakes, all-bran, white rice, whole grain rice, pasta, etc | whole grain bread, non-wholegrain bread, porridge, whole grain cereals, non-wholegrain cereals | white bread, brown bread, wheat bread, whole wheat bread, multigrain bread, other bread, croissant, white buns, currant bun, white rusk, brown buns (with muesli), crispbread, whole-wheat rusk, whole-wheat crispbread, rye bread, other bread substitutes, gingerbread (loaf), granola, cornflakes, fibre-rich breakfast product, rice, noodles |
| Dairy | g/d | 20+/30+ cheese, 40+ cheese, 48+ cheese, cream cheese/foreign cheese, full-fat milk, semi-skimmed milk, skimmed milk, butter milk, other dairy beverages, full-fat custard, full-fat yoghurt, skimmed yoghurt, skimmed fruit yoghurt, cottage cheese/fruit cottage cheese, other types of dairy, semi-skimmed coffee milk, full-fat coffee milk, semi-skimmed milk used in coffee, other types of milk used in coffee, cheese eaten with warm meal, cheese eaten as snack, butter | semi-skimmed milk, low-fat milk, butter milk, full-fat yoghurt, full-fat custard, 20+/30+ cheese, 40+ cheese, 48+ cheese, full-fat luxury cheese, semi-fat luxury cheese, unknown type of cheese, skimmed milk/butter milk eaten with breakfast cereals, semi-skimmed milk eaten with breakfast cereals, full-fat milk eaten with breakfast cereals, low-fat (fruit) yoghurt eaten with breakfast cereals, semi-skimmed (fruit) yoghurt eaten with breakfast cereals, full-fat (fruit) yoghurt eaten with breakfast cereals, (fruit) cottage cheese eaten with breakfast cereals, cheese eaten as snack, cheese eaten with warm meal, cream eaten with warm meal, full-fat milk, unknown type of milk, semi-skimmed yoghurt, low-fat yoghurt, unknown type of yoghurt/custard, semi-skimmed coffee milk, diet coffee milk, full-fat milk, semi-skimmed milk, unknown type of coffee milk, butter, semi-skimmed butter | whole milk, low-fat milk, non-fat milk, condensed milk, cream, single cream, milkshakes, milk based drinks, yogurt whole, yogurt nonfat, petit suisse, cheese fresh (cottage cheese), cheese in portions or spreadable cheese, other cheese i.e cured, semicured (manchego, emmenthal, edam, etc.), cheese fresh white (burgos, goat...), butter | dairy low fat, dairy full fat | 20% fat cheese, 30% fat cheese, regular cheese, full-fat luxury cheese, reduced fat luxury cheese, unknown type of cheese, other cheese, full-fat milk, low-fat/semi-skimmed milk, skimmed milk, buttermilk, other types of milk, full-fat yoghurt, custard etc. , skimmed yoghurt, custard etc., other yoghurt, custard etc. , low fat/semi-skimmed coffee milk, regular full fat milk in coffee, regular low fat/semi-skimmed milk in coffee, regular skimmed milk in coffee, unknown coffee milk, other coffee milk, cheese with the warm meal, cubes of cheese, butter, low-fat butter, butter/ low-fat butter |
| Meat | g/d | cold meats/sausage, other type of cold cuts, minced meat, smoked sausage, roast beef/steak/etc, beef streaked/other types of beef, pork schnitzel, pork chops, bacon, chicken, other types of meat or poultry, cold meats or sausage eaten as snack | minced meat, chicken/turkey, beef: steak, beef: tender stead, pork chops, pork filet, smoked sausage, other meats, liver sausage, other type of sausage, liver or kidney products, warm deep-fried snacks eaten with warm meal, unknown type of meat, boiled liver, liver products, ham, colt meats/sausage, bacon, unknown type of cold meats | chicken or turkey with skin, chicken or turkey without skin, beef or veal, pork, lamb, rabbit or hare, liver (beef, pork, chicken), other organ meats (brain, heart, gizzards), cured ham, cooked ham, processed meats (salami, chorizo, morcilla, bologna, sausages, butifarra, sobrasada), pâtés, foie gras, hamburger, meatballs, bacon, pork belly | red meat and processed meat (e.g. pork, beef, veal, lamb, hamburger or sausages), white meat (e.g. poultry, rabbit) | cooked liver, liver products, gammon, lucheon meat, bacon, unknown types of meat, other type of meat, steak etc., blind finch etc., gamon steak, pork fillet, hamburgee etc., chicken/turkey without skin etc., chicken with skin etc., minced meat., liver and kidney products, other kind of meat, snack: meatball ect., snack: satay, snack: sausage roll ect., sausage |
| Fats and oils | g/d | margarine or butter, halvarine, other types of fat spreads | halvarine, diet halvarine, cholesterol-reducing halvarine, margarine (semi-solid), margarine (solid), diet margarine, cholesterol-reducing margarine, liquid margarine, solid frying fat, liquid frying fat, solid deep-frying fat, liquid deep-frying fat, lard, olive oil, oil rich in MUFA, oil rich in PUFA, unknown type of fat spread, other type of fat spread, unknown type of fat used for food preparation | olive oil refined, olive oil extra virgin, olive-pomace oil, corn oil, sunflower seed oil, soybean oil, mix of previous oils, margarine, pork lard | na | low-fat margarine, low-fat diet margarine, cholesterol-lowering low-fat margarine, tub margarine, stick margarine, diet margarine, cholesterol-lowering margarine, liquid margarine, solid bake and frying fat, liquid bake and frying fat, other types of oil, solid deep frying oil, liquid deep frying oil, lard, olive oil, deep frying oil |
| Sugary snacks | g/d | candy bars, candy, small cookies, biscuits, cake, large cookies, pastry and pies, nutritional biscuits, chocolate milk, chocolate sandwich topping, sweet sandwich toppings, apple sauce | candy bar, candy, small cookies, biscuits, cake, large cookies, nutritional biscuits, pastry and pies, milk chocolate, white chocolate, chocolate-based sandwich topping, sweet sandwich topping | baked goods, donuts, muffins, pastries, churros, fritters, other fried dough, almond nougat (turrón), mantecados (lard shortbread), marzipan  chocolates and bonbons  jam, marmalade | candies, sweet snacks (sweets, biscuits, ice cream, cakes, pastries) | sweets/candies, candy bars  biscuits and cookies, large cookies, pie or cake, food rusk, chocolate spread, milk chocolate, white chocolate, coconut topping (bread), sweet toppings, apple sauce, rosehip syrup, pudding |
| Legumes | g/d | legumes, soup with legumes | legumes, unknown type of vegetarian products, soy milk eaten with breakfast cereals, vegetarian cold meats, soy milk, soy drink, soy dessert, soup with legumes, tofu/tahoe, other vegetarian products | green beans, lentils, beans (pinto, white or black) chickpeas peas, fava beans | lentils, beans, peas | soup with legumes, peas, broad beans, green beans, legumes, vegetarian burger, soy products: tahoe/tofu, tempeh, other meat substitutes |
| Potatoes | g/d | boiled/mashed potatoes, french fries/baked potatoes | boiled/mashed potatoes, fried potatoes / mashed potatoes with added fat, french fries prepared in oven, french fries home deep-fried, french fries not prepared at home, french fries eaten as snack | potato chips/crisps, commercial, french fries, homemade, potato, roasted or boiled | na | boiled potatoes, mashed potatoes, baked potatoes, other potatoes, oven fries, fried fries, fries unknown preparation, snack: fries |
| Nuts | g/d | peanut butter, peanuts or nuts eaten with warm meal, peanuts and nuts as snack | peanuts, walnuts, mixed nuts and raisins, other types of nuts, line seed, peanut butter, nuts and seeds eaten with warm meal, peanut sauce | almonds, pistachios, walnuts, other nuts | nuts or seeds | peanut butter, nuts and seeds with the warm meal, satay - or peanut sauce, nuts |
| Coffee | g/d | coffee | black coffee | coffee, decaffeinated coffee | coffee | coffee with caffeine, coffee without caffeine, cappuccino |
| Tea | g/d | tea | black tea, green tea, herbal tea | tea | tea | black tea, green tea, herbal tea, other types of tea |
| Juice | g/d | fruit juice and fruit drinks | orange juice, other types of fruit juice, vegetable juice | orange juice, fresh squeezed, other fruit juices, fresh squeezed, fruit juice, commercial | juice with sugar, juice without sugar | orange juice, apple juice, grapefruit juice, tomato juice, vegetable juice, other juice |
| SSBs | g/d | soft drinks/lemonades with sugar | soft drink | soft drinks with sugar | soft drinks with sugar | coke with caffeine, other soda, ice tea |
| LNCBs | g/d | light soft drinks/lemonade without sugar | light soft drinks | light soft drinks | soft drink without sugar | na |
| Water | g/d | water | mineral water | tap and bottled water | water | na |
| AOC: Alpha Omega Cohort; LNCBs: Low/no-calorie beverages; MET: Metabolic equivalent of task; NA: Not available; SSBs: Sugar-sweetened beverages. | | | | | | |

Supplemental Table 3. Baseline characteristics of the EU prospective cohort studies by category of sugar-sweetened beverages.

|  | Lifelines | | | x | NQplus | | | x | Predimed-Plus | | | x | Feel4diabetes | | | x | AOC | | |
| --- | --- | --- | --- | --- | --- | --- | --- | --- | --- | --- | --- | --- | --- | --- | --- | --- | --- | --- | --- |
|  | No of servings/week | | |  | No of servings/week | | |  | No of servings/week | | |  | No of servings/week | | |  | No of servings/week | | |
| Characteristics ^a^ | 0 | ≤ 2 | > 2 |  | 0 | ≤ 2 | > 2 |  | 0 | ≤ 2 | > 2 |  | 0 | ≤ 2 | > 2 |  | 0 | ≤ 2 | > 2 |
| N | 29,637 | 20,207 | 28,442 |  | 817 | 434 | 193 |  | 156 | 44 | 15 |  | 328 | 57 | 96 |  | 1085 | 463 | 745 |
| Women, % | 70 | 59 | 50* |  | 58 | 39 | 23* |  | 73 | 61 | 33* |  | 69 | 56 | 50* |  | 28 | 16 | 13* |
| Age, years | 51 | 46 | 41* |  | 56 | 52 | 49* |  | 65 | 66 | 62* |  | 44 | 43 | 42 |  | 69 | 68 | 68* |
| Education, % |  |  |  |  |  |  |  |  |  |  |  |  |  |  |  |  |  |  |  |
| -low | 5 | 4 | 3* |  | 1 | 1 | 1 |  | 70 | 80 | 67 |  | 8 | 9 | 9 |  | 58 | 53 | 58 |
| -medium | 64 | 61 | 68 |  | 43 | 43 | 53 |  | 26 | 21 | 33 |  | 54 | 53 | 63 |  | 31 | 34 | 226 |
| -high | 31 | 36 | 28 |  | 57 | 56 | 47 |  | 5 | 0 | 0 |  | 38 | 39 | 28 |  | 12 | 13 | 12 |
| Body weight, kg | 79.2 | 78.1 | 80.9* |  | 77.6 | 80.0 | 82.7* |  | 84.1 | 83.3 | 88.1 |  | 80.2 | 80.4 | 81.9 |  | 81.1 | 81.8 | 84.2* |
| Waist circumference, cm | 90.8 | 88.9 | 90.1* |  | 90.8 | 91.8 | 93.5* |  | 106.9 | 105.5 | 107.4 |  | 93.8 | 94.9 | 95.7 |  | 100.6 | 100.9 | 102.6* |
| BMI, kg/m² | 26.5 | 25.5 | 25.8* |  | 25.9 | 25.9 | 25.9 |  | 32.4 | 32.2 | 31.7 |  | 28.8 | 28.7 | 28.5 |  | 27.6 | 27.5 | 27.9 |
| BMI categories |  |  |  |  |  |  |  |  |  |  |  |  |  |  |  |  |  |  |  |
| -normal | 40 | 50 | 47* |  | 47 | 45 | 46 |  | 0 | 0 | 0 |  | 27 | 33 | 29 |  | 24 | 21 | 20 |
| -overweight | 42 | 39 | 39 |  | 38 | 42 | 42 |  | 31 | 27 | 40 |  | 38 | 33 | 38 |  | 53 | 58 | 55 |
| -obese | 18 | 11 | 14 |  | 14 | 13 | 12 |  | 69 | 73 | 60 |  | 35 | 33 | 33 |  | 23 | 21 | 25 |
| **Lifestyle** |  |  |  |  |  |  |  |  |  |  |  |  |  |  |  |  |  |  |  |
| Physical activity (METs-min per week, min/week or %) ^b^ |  |  |  |  |  |  |  |  |  |  |  |  |  |  |  |  |  |  |  |
| -intense | 0 | 0 | 0* |  | 420 | 399 | 525 |  | 154 | 294 | 104 |  | 0 | 0 | 120* |  | 37 | 42 | 40 |
| -moderate | 1780 | 1680 | 1520* |  | 815 | 810 | 764 |  | 385 | 542 | 839 |  | 30 | 90 | 120* |  | 21 | 22 | 21 |
| -light | 0 | 0 | 0* |  | 0 | 0 | 0 |  | 336 | 559 | 210 |  | 180 | 180 | 210 |  | 37 | 32 | 34 |
| Sedentary, min/week or % ^c^ | 1050 | 840 | 980* |  | 1980 | 1830 | 1860 |  | 1740 | 1710 | 2100 |  | 1680 | 2520 | 1260* |  | 6 | 4 | 5 |
| Smoking, % |  |  |  |  |  |  |  |  |  |  |  |  |  |  |  |  |  |  |  |
| -never | 49 | 49 | 51* |  | 49 | 55 | 56* |  | 69 | 73 | 60 |  | 47 | 42 | 33* |  | 20 | 18 | 12* |
| -former | 44 | 35 | 26 |  | 45 | 37 | 33 |  | 27 | 25 | 33 |  | 26 | 25 | 20 |  | 65 | 68 | 71 |
| -current | 16 | 16 | 23 |  | 6 | 8 | 11 |  | 5 | 2 | 7 |  | 27 | 33 | 47 |  | 15 | 15 | 17 |
| Alcohol intake, % ^d^ |  |  |  |  |  |  |  |  |  |  |  |  |  |  |  |  |  |  |  |
| -none | 4 | 1 | 2* |  | 5 | 3 | 5 |  | 46 | 41 | 27 |  | 48 | 22 | 34* |  | 6 | 2 | 3* |
| -low | 69 | 72 | 73 |  | 53 | 59 | 57 |  | 44 | 48 | 47 |  | 19 | 32 | 17 |  | 53 | 52 | 52 |
| -medium | 20 | 20 | 18 |  | 20 | 19 | 24 |  | 11 | 11 | 27 |  | 18 | 19 | 21 |  | 17 | 23 | 21 |
| -high | 7 | 7 | 7 |  | 22 | 19 | 15 |  | 0 | 0 | 0 |  | 15 | 28 | 28 |  | 24 | 23 | 24 |
| **Dietary intake** |  |  |  |  |  |  |  |  |  |  |  |  |  |  |  |  |  |  |  |
| Total energy, kcal/d | 1782 | 1985 | 2195* |  | 1893 | 2078 | 2421* |  | 2016 | 2118 | 2553* |  | NA | NA | NA |  | 1804 | 1870 | 1956* |
| Fruit, g/d | 152 | 110 | 85* |  | 220 | 163 | 102* |  | 356 | 439 | 311 |  | 71 | 45 | 45* |  | 120 | 117 | 108* |
| Vegetables, g/d | 111 | 109 | 76* |  | 156 | 140 | 128* |  | 268 | 252 | 250 |  | 196 | 125 | 161 |  | 75 | 73 | 71* |
| Grains, g/d | 166 | 186 | 189* |  | 174 | 194 | 214* |  | 105 | 112 | 101 |  | 120 | 165 | 120 |  | 164 | 170 | 171 |
| Dairy, g/d | 273 | 280 | 257* |  | 284 | 292 | 249* |  | 440 | 382 | 591 |  | 120 | 180 | 120 |  | 241 | 264 | 224 |
| Meat, g/d | 72 | 75 | 83* |  | 64 | 71 | 81* |  | 101 | 98 | 124 |  | 94 | 118 | 122* |  | 80 | 78 | 81 |
| Fats, g/d | 20 | 23 | 24* |  | 23 | 27 | 32* |  | 20 | 22 | 15 |  | NA | NA | NA |  | 31 | 34 | 36* |
| Sugary snacks, g/d | 53 | 65 | 73* |  | 37 | 46 | 61* |  | 47 | 38 | 78 |  | 11 | 20 | 20* |  | 59 | 65 | 67* |
| Legumes, g/d | 11 | 15 | 11* |  | 38 | 40 | 36 |  | 50 | 54 | 50 |  | 57 | 86 | 71 |  | 21 | 22 | 23 |
| Potatoes, g/d | 74 | 88 | 96* |  | 50 | 70 | 80* |  | 50 | 82 | 111* |  | NA | NA | NA |  | 99 | 99 | 99 |
| Nuts, g/d | 7 | 8 | 8* |  | 12 | 12 | 11 |  | 13 | 7 | 17 |  | 6 | 2 | 6 |  | 3 | 4 | 3* |
| Coffee, g/d | 465 | 465 | 402* |  | 406 | 406 | 406* |  | 50 | 88 | 100 |  | 286 | 268 | 339 |  | 375 | 375 | 375 |
| Tea, g/d | 232 | 232 | 161* |  | 174 | 174 | 121* |  | 0 | 0 | 0 |  | 0 | 0 | 0 |  | 150 | 150 | 150 |
| Juice, g/d | 13 | 27 | 43* |  | 21 | 27 | 43* |  | 42 | 86 | 86 |  | 36 | 71 | 71 |  | 54 | 54 | 62 |
| LNCBs, servings/d | 0 | 0 | 0* |  | 0 | 0 | 0* |  | 0 | 0 | 0* |  | 0 | 0 | 0* |  | NA | NA | NA |
| Water, servings/d | 3 | 2 | 2* |  | 0 | 0 | 0* |  | 9 | 6 | 6* |  | 8 | 7 | 7 |  | NA | NA | NA |
| **History of diseases, %** |  |  |  |  |  |  |  |  |  |  |  |  |  |  |  |  |  |  |  |
| Diabetes | 4 | 1 | 1* |  | 4 | 3 | 1 |  | 36 | 16 | 27 |  | 20 | 14 | 19 |  | 17 | 12 | 13* |
| CVD | 3 | 2 | 2* |  | 3 | 3 | 2 |  | 11 | 30 | 13* |  | NA | NA | NA |  | 100 | 100 | 100 |
| Hypertension | 28 | 21 | 18* |  | 27 | 23 | 20 |  | 90 | 98 | 73* |  | 24 | 28 | 30 |  | 50 | 52 | 47 |
| Hypercholesterolemia | 19 | 13 | 10* |  | 21 | 18 | 16 |  | 89 | 84 | 80 |  | 6 | 7 | 8 |  | NA | NA | NA |
| Cancer | 7 | 5 | 3* |  | 6 | 5 | 3 |  | 6 | 2 | 0 |  | NA | NA | NA |  | 11 | 8 | 10 |
| **Outcomes** |  |  |  |  |  |  |  |  |  |  |  |  |  |  |  |  |  |  |  |
| Body weight change, kg/y | -0.01 | -0.02 | 0.08* |  | -0.17 | -0.41 | 0.15 |  | -0.98 | -1.01 | 0.16 |  | 0.23 | -0.06 | -0.21 |  | -0.04 | -0.04 | -0.05 |
| Waist circumference change, cm/y | 0.01 | -0.03 | 0.04* |  | 0.26 | 0.07 | 0.13 |  | -0.82 | -0.61 | -0.77 |  | 0.19 | -0.19 | -0.03 |  | 0.09 | 0.01 | 0.06 |
| ^a^ Mean, Median, or %. Differences across categories of SSB consumption were assessed using ANOVA or Kruskal-Wallis for continuous variables and Chi-square tests for categorical variables. P-values <0.05 are indicated with an * in the third intake column (and upper category for categorical variables) of each cohort where appropriate.  ^b^ METs-min per week in Lifelines, NQplus, and Predimed-Plus; min/week in the Feel4diabetes study and categorical in the Alpha Omega Cohort: light (>0-3 METs), moderate (>0 to <5 days/week of moderate or vigorous activity, >3 METs) or high (≥5 days/week of moderate or vigorous activity).  ^c^ min/week or % of participants with no activity in the Alpha Omega Cohort.  ^d^ alcohol intake is expressed as g/d of ethanol for all cohorts and categorized into none (0 g/d), low (>0–10 g/d), medium (>10–20 g/d), or high (>20 g/d), except in the Feel4diabetes study where categorization was: no alcoholic beverages (0g), 1-50g, 50-95g, and >95g.  AOC: Alpha Omega Cohort. BMI: Body Mass Index. CVD: Cardiovascular diseases. LNCBs: Low/no-calorie beverages. MET: METabolic equivalent of task. NA: Not available. | | | | | | | | | | | | | | | | | | | |

Supplemental Table 4. Baseline characteristics of the EU prospective cohort studies by category of low/no-calorie beverages.

|  | Lifelines | | |  | NQplus | | |  | Predimed-Plus | | |  | Feel4diabetes | | |
| --- | --- | --- | --- | --- | --- | --- | --- | --- | --- | --- | --- | --- | --- | --- | --- |
|  | No of servings/week | | |  | No of servings/week | | |  | No of servings/week | | |  | No of servings/week | | |
| Characteristics ^a^ | 0 | ≤ 2 | > 2 |  | 0 | ≤ 2 | > 2 |  | 0 | ≤ 2 | > 2 |  | 0 | ≤ 2 | > 2 |
| N | 33,938 | 16,599 | 27,749 |  | 973 | 276 | 195 |  | 160 | 40 | 15 |  | 314 | 62 | 105 |
| Women, % | 58 | 63 | 60* |  | 51 | 44 | 35* |  | 65 | 80 | 67 |  | 65 | 66 | 59 |
| Age, years | 47 | 47 | 43* |  | 54 | 53 | 52 |  | 65 | 64 | 64 |  | 44 | 42 | 43 |
| Education, % |  |  |  |  |  |  |  |  |  |  |  |  |  |  |  |
| -low | 4 | 4 | 4* |  | 1 | 1 | 0 |  | 71 | 78 | 67 |  | 10 | 2 | 8 |
| -medium | 65 | 62 | 67 |  | 42 | 49 | 50 |  | 26 | 23 | 27 |  | 53 | 63 | 59 |
| -high | 31 | 35 | 30 |  | 58 | 50 | 50 |  | 4 | 0 | 7 |  | 37 | 36 | 33 |
| **Anthropometrics** |  |  |  |  |  |  |  |  |  |  |  |  |  |  |  |
| Body weight, kg | 77.9 | 78.2 | 82.3* |  | 77.4 | 80.7 | 84.8* |  | 83.0 | 89.3 | 84.0* |  | 78.4 | 81.2 | 86.6* |
| Waist circumference, cm | 89.1 | 89.2 | 91.8* |  | 90.1 | 93.0 | 95.7* |  | 105.8 | 110.2 | 106.1* |  | 93.0 | 93.7 | 98.6* |
| BMI, kg/m² | 25.5 | 25.8 | 26.8* |  | 25.5 | 26.3 | 27.3* |  | 32.0 | 33.8 | 31.9* |  | 28.1 | 29.0 | 30.5* |
| Categories BMI |  |  |  |  |  |  |  |  |  |  |  |  |  |  |  |
| -normal weight | 50 | 47 | 38* |  | 51 | 40 | 33* |  | 0 | 0 | 0 |  | 33 | 24 | 16* |
| -overweight | 38 | 41 | 42 |  | 39 | 44 | 42 |  | 34 | 15 | 40 |  | 36 | 39 | 41 |
| -obese | 12 | 13 | 20 |  | 11 | 16 | 25 |  | 66 | 85 | 60 |  | 31 | 37 | 43 |
| **Lifestyle** |  |  |  |  |  |  |  |  |  |  |  |  |  |  |  |
| Physical activity (METs/min-week or min/week) ^b^ |  |  |  |  |  |  |  |  |  |  |  |  |  |  |  |
| -intense | 0 | 0 | 0* |  | 420 | 280 | 630 |  | 168 | 168 | 112 |  | 0 | 0 | 60 |
| -moderate | 1647 | 1767 | 1618* |  | 788 | 840 | 834 |  | 559 | 175 | 0 |  | 50 | 60 | 60 |
| -light | 0 | 0 | 0 |  | 0 | 0 | 0 |  | 336 | 277 | 373 |  | 180 | 210 | 210 |
| Sedentary, min/week or % ^c^ | 840 | 840 | 1050* |  | 1860 | 1980 | 1860 |  | 1770 | 1680 | 1800 |  | 1680 | 1680 | 1680 |
| Smoking |  |  |  |  |  |  |  |  |  |  |  |  |  |  |  |
| -never | 45 | 48 | 47* |  | 53 | 49 | 47 |  | 68 | 75 | 60 |  | 43 | 40 | 45 |
| -former | 36 | 37 | 33 |  | 40 | 42 | 45 |  | 28 | 23 | 33 |  | 24 | 29 | 26 |
| -current | 19 | 15 | 20 |  | 7 | 10 | 8 |  | 4 | 3 | 7 |  | 33 | 31 | 30 |
| Alcohol intake, % ^d^ |  |  |  |  |  |  |  |  |  |  |  |  |  |  |  |
| -none | 3 | 2 | 2* |  | 5 | 3 | 4 |  | 43 | 55 | 20 |  | 41 | 41 | 47 |
| -low | 70 | 73 | 73 |  | 55 | 58 | 54 |  | 44 | 40 | 67 |  | 22 | 20 | 17 |
| -medium | 20 | 20 | 18 |  | 20 | 23 | 21 |  | 14 | 5 | 13 |  | 19 | 20 | 16 |
| -high | 8 | 6 | 6 |  | 21 | 16 | 22 |  | 0 | 0 | 0 |  | 19 | 20 | 21 |
| **Dietary intake** |  |  |  |  |  |  |  |  |  |  |  |  |  |  |  |
| Total energy, kcal/d | 1992 | 1915 | 1996* |  | 1977 | 2009 | 2060* |  | 2122 | 1854 | 2140* |  | NA | NA | NA |
| Fruits, g/d | 110 | 152 | 110* |  | 215 | 212 | 158* |  | 361 | 343 | 453 |  | 45 | 71 | 71 |
| Vegetables, g/d | 108 | 109 | 107* |  | 150 | 141 | 136* |  | 258 | 249 | 287 |  | 196 | 196 | 161 |
| Grains, g/d | 180 | 180 | 179* |  | 184 | 194 | 190 |  | 105 | 98 | 109 |  | 120 | 120 | 150 |
| Dairy, g/d | 263 | 287 | 263* |  | 283 | 285 | 266 |  | 529 | 348 | 612* |  | 120 | 240 | 120 |
| Meat, g/d | 74 | 74 | 80* |  | 64 | 75 | 80* |  | 106 | 84 | 120* |  | 94 | 94 | 118 |
| Fats, g/d | 23 | 22 | 22* |  | 24 | 26 | 28* |  | 21 | 19 | 20 |  | NA | NA | NA |
| Sugary snacks, g/d | 61 | 62 | 66* |  | 41 | 44 | 48* |  | 43 | 52 | 67 |  | 20 | 20 | 20 |
| Legumes, g/d | 11 | 15 | 11* |  | 39 | 37 | 36* |  | 50 | 52 | 44 |  | 57 | 71 | 57 |
| Potatoes, g/d | 88 | 86 | 88* |  | 57 | 72 | 66* |  | 78 | 50 | 50 |  | NA | NA | NA |
| Nuts, g/d | 8 | 8 | 8 |  | 12 | 11 | 13 |  | 13 | 6 | 13 |  | 2 | 6 | 6 |
| Coffee, g/d | 465 | 465 | 465* |  | 406 | 406 | 406 |  | 50 | 50 | 125 |  | 321 | 250 | 286 |
| Tea, g/d | 232 | 232 | 161* |  | 174 | 174 | 174* |  | 0 | 0 | 0 |  | 0 | 0 | 0* |
| Juice, g/d | 22 | 22 | 27* |  | 21 | 27 | 27* |  | 86 | 29 | 29 |  | 36 | 71 | 54 |
| SSBs, servings/d | 0 | 0 | 0* |  | 0 | 0 | 0* |  | 0 | 0 | 0* |  | 0 | 0 | 0* |
| Water, servings/d | 2 | 2 | 2* |  | 0 | 0 | 0* |  | 6 | 6 | 9 |  | 8 | 8 | 7* |
| **History of diseases, %** |  |  |  |  |  |  |  |  |  |  |  |  |  |  |  |
| Self-reported diabetes | 2 | 2 | 3* |  | 3 | 4 | 5 |  | 29 | 35 | 47 |  | 19 | 26 | 17 |
| CVD | 2 | 2 | 2 |  | 2 | 4 | 5* |  | 18 | 10 | 0 |  | NA | NA | NA |
| Hypertension | 21 | 23 | 23* |  | 23 | 28 | 28 |  | 89 | 93 | 100 |  | 25 | 26 | 30 |
| Hypercholesterolemia | 14 | 15 | 14* |  | 18 | 21 | 24 |  | 86 | 90 | 100 |  | 7 | 8 | 5 |
| Cancer | 5 | 5 | 4* |  | 5 | 5 | 6 |  | 3 | 12 | 0 |  | NA | NA | NA |
| **Outcomes** |  |  |  |  |  |  |  |  |  |  |  |  |  |  |  |
| Body weight change, kg/y | 0.02 | -0.01 | 0.05* |  | -0.20 | -0.11 | -0.33 |  | -0.88 | -1.26 | -0.15 |  | 0.34 | -0.57 | -0.17 |
| Waist circumference change, cm/y | 0.01 | 0.0 | 0.01 |  | 0.26 | 0.15 | -0.16 |  | -0.85 | -0.84 | 0.20 |  | 0.25 | -0.27 | -0.13 |
| ^a^ Mean, Median, or %. Differences across categories of LNCB consumption were assessed using ANOVA or Kruskal-Wallis for continuous variables and Chi-square tests for categorical variables. P-values <0.05 are indicated with an * in the third intake column (and upper category for categorical variables) of each cohort where appropriate.  ^b^ METs-min per week in Lifelines, NQplus, and Predimed-Plus; min/week in the Feel4diabetes study and categorical in the Alpha Omega Cohort: light (>0-3 METs), moderate (>0 to <5 days/week of moderate or vigorous activity, >3 METs) or high (≥5 days/week of moderate or vigorous activity).  ^c^ min/week or % of participants with no activity in the Alpha Omega Cohort.  ^d^ alcohol intake is expressed as g/d of ethanol for all cohorts and categorized into none (0 g/d), low (>0–10 g/d), medium (>10–20 g/d), or high (>20 g/d), except in the Feel4diabetes study where categorization was: no alcoholic beverages (0g), 1-50g, 50-95g, and >95g.  AOC: Alpha Omega Cohort. BMI: Body Mass Index. CVD: Cardiovascular diseases. MET: METabolic equivalent of task. NA: Not available. SSBs: Sugar-sweetened beverages. | | | | | | | | | | | | | | | |

|  |  |  |  |  |  |  | Meta-analyses | |
| --- | --- | --- | --- | --- | --- | --- | --- | --- |
| Variables | Lifelines  β (SE) | NQplus  β (SE) | Predimed-Plus  β (SE) | Feel4Diabetes  β (SE) | AOC  β (SE) |  | Random-effects estimate  (β 95%CI) | Heterogeneity  I^2^, P-value |
| N | 78,286 | 1,444 | 215 | 481 | 2,293 |  |  |  |
| *SSB* |  |  |  |  |  |  |  |  |
| Body weight change, kg/y | | | | | | | | |
| model 1 | 0.03 (0.01) | 0.09 (0.15) | 0.60 (0.85) | 0.44 (0.22) | -0.002 (0.03) |  | 0.03 [0.01; 0.05] | 20.7%, P = 0.28 |
| model 2 | 0.03 (0.01) | 0.10 (0.15) | 0.45 (0.84) | 0.42 (0.22) | 0.01 (0.03) |  | 0.03 [0.01; 0.05] | 1.1%, P = 0.40 |
| model 3 | 0.02 (0.01) | 0.08 (0.15) | 0.55 (0.89) | 0.39 (0.24) | 0.01 (0.03) |  | 0.02 [0.00; 0.04] | 0%, P = 0.56 |
| Waist circumference change, cm/y | |  |  |  |  |  |  |  |
| model 1 | 0.03 (0.01) | -0.37 (0.20) | -0.18 (1.02) | 0.45 (0.25) | 0.05 (0.05) |  | 0.03 [0.01; 0.05] | 43.0%, P = 0.13 |
| model 2 | 0.05 (0.01) | -0.32 (0.20) | -0.70 (0.95) | 0.45 (0.25) | 0.07 (0.04) |  | 0.05 [0.03; 0.07] | 41.6%, P = 0.14 |
| model 3 | 0.03 (0.01) | -0.37 (0.20) | -0.51 (1.05) | 0.33 (0.26) | 0.07 (0.04) |  | 0.03 [0.01; 0.05] | 39%, P = 0.16 |
| *LNCB* |  |  |  |  |  |  |  |  |
| Body weight change, kg/y | | | | | | | | |
| model 1 | 0.01 (0.01) | -0.02 (0.16) | 0.20 (0.52) | -0.47 (0.26) | - |  | 0.01 [-0.01; 0.03] | 16%, P = 0.31 |
| model 2 | 0.07 (0.01) | 0.08 (0.16) | 0.20 (0.51) | -0.39 (0.27) | - |  | 0.07 [0.05; 0.09] | 0%, P = 0.40 |
| model 3 | 0.06 (0.01) | 0.10 (0.16) | -0.04 (0.53) | -0.38 (0.27) | - |  | 0.06 [0.04; 0.08] | 0%, P = 0.43 |
| model 4 | 0.06 (0.01) | 0.09 (0.16) | -0.04 (0.53) | *model 3 used | - |  | 0.06 [0.04; 0.08] | 0%, P = 0.44 |
| Waist circumference change, cm/y | |  |  |  |  |  |  |  |
| model 1 | 0.02 (0.01) | -0.33 (0.22) | 0.36 (0.62) | -0.49 (0.29) | - |  | -0.14 [-0.43; 0.15] | 49%, P = 0.12 |
| model 2 | 0.13 (0.01) | -0.01 (0.21) | 0.46 (0.58) | -0.36 (0.30) | - |  | 0.09 [-0.06; 0.24] | 13%, P = 0.33 |
| model 3 | 0.12 (0.01) | 0.05 (0.22) | 0.08 (0.62) | -0.38 (0.30) | - |  | 0.08 [-0.05; 0.22] | 0%, P = 0.41 |
| model 4 | 0.12 (0.01) | 0.04 (0.22) | 0.08 (0.62) | *model 3 used | - |  | 0.08 [-0.07; 0.23] | 0%, P = 0.41 |
| Models were adjusted for age, sex, and intervention group (Feel4diabetes, Predimed-Plus and AOC) (model 1), baseline weight or waist circumference and height (model 2), education, physical activity, sedentary behaviour, alcohol intake, smoking; all dietary data (model 3) and total energy intake (model 4; expect Feel4diabetes).  AOC: Alpha Omega Cohort. CI: Confidence Interval. SE: Standard Error. | | | | | | | | |

Supplemental Table 5. Meta-analyses of sugar-sweetened beverages (SSBs) and low/non-calorie beverages (LNCBs) with yearly body weight and waist circumference change in the EU prospective cohort studies.

Supplemental Table 6. Meta-analyses of the associations between the theoretical substitution of one serving beverage for another and yearly body weight and waist circumference change in the EU prospective cohort studies.

|  |  |  |  |  |  | Meta-analyses | |
| --- | --- | --- | --- | --- | --- | --- | --- |
| Models | Lifelines  β (SE) | NQplus  β (SE) | Predimed-Plus  β (SE) | Feel4Diabetes  β (SE) |  | Random-effects estimate  (β 95%CI) | Heterogeneity  I^2^, P-value |
| *LNCB for SSB* |  |  |  |  |  |  |  |
| N | 78,286 | 1444 | 215 | 481 |  |  |  |
| Body weight change, kg/y | 0.04 (0.01) | 0.03 (0.22) | -0.59 (0.98) | -0.77 (0.35) |  | -0.14 [-0.51; 0.24] | 48%, P = 0.12 |
| Waist circumference change, cm/y | 0.09 (0.01) | 0.42 (0.30) | 0.59 (1.15) | -0.70 (0.39) |  | 0.04 [-0.42; 0.49] | 45%, P = 0.14 |
| *Water for SSB* |  |  |  |  |  |  |  |
| N | 22,859 | 1444 | 156 | 481 |  |  |  |
| Body weight change, kg/y | -0.02 (0.01) | 0.35 (0.25) | -0.50 (1.09) | -0.41 (0.24) |  | -0.04 [-0.35; 0.27] | 40%, P = 0.17 |
| Waist circumference change, cm/y | -0.03 (0.02) | 0.96 (0.34) | 1.14 (1.44) | -0.33 (0.27) |  | 0.20 [-0.46; 0.87] | 71%, P = 0.02 |
| *Water for LNCB* |  |  |  |  |  |  |  |
| N | 22,859 | 1444 | 156 | 481 |  |  |  |
| Body weight change, kg/y | -0.05 (0.01) | 0.34 (0.27) | 0.07 (0.59) | 0.41 (0.27) |  | 0.13 [-0.17; 0.42] | 40%, P = 0.17 |
| Waist circumference change , cm/y | **-**0.08 (0.02) | 0.58 (0.36) | 0.33 (0.78) | 0.39 (0.30) |  | 0.19 [-0.21; 0.58] | 50%, P = 0.11 |
| Models were adjusted for age, sex, and intervention group (Feel4diabetes, Predimed-Plus), baseline weight or waist circumference and height , education, physical activity, sedentary behaviour, alcohol intake, smoking; all dietary data and total energy intake (only models of water as substitute for LNCB; except Feel4diabetes where total energy was not available).  CI: Confidence Interval. LNCB: Low/no-calorie beverage. SE: Standard Error. SSB: Sugar-sweetened beverage. | | | | | | | |

Supplemental Table 7. Meta-analyses of sugar-sweetened beverages (SSBs) and low/non-calorie beverages (LNCBs) with yearly body weight and waist circumference change stratified by BMI and sex in the EU prospective cohort studies.

|  |  |  |  |  |  |  | Meta-analyses | |
| --- | --- | --- | --- | --- | --- | --- | --- | --- |
| Models | Lifelines  β (SE) | NQplus  β (SE) | Predimed-Plus  β (SE) | Feel4Diabetes  β (SE) | AOC  β (SE) |  | Random-effects estimate  β (95%CI) | Heterogeneity  I^2^, P-value |
| *SSB* |  |  |  |  |  |  |  |  |
| Body weight change, kg/y |  |  |  |  |  |  |  |  |
| BMI < 25 kg/m^2^ | 0.04 (0.01)* | 0.17 (0.20) | - | -0.39 (0.59) | 0.13 (0.07)* |  | 0.06 [-0.01; 0.12] | 0%, P = 0.46 |
| BMI ≥ 25 kg/m^2^ | 0.00 (0.01) | 0.04 (0.22) | 0.55 (0.89) | 0.45 (0.28) | -0.02 (0.04) |  | -0.00 [-0.02; 0.02] | 0%, P = 0.52 |
| Men | 0.02 (0.01) | 0.03 (0.18) | 0.93 (1.52) | 0.64 (0.56) | 0.00 (0.04) |  | 0.02 [0.00; 0.04] | 0%, P = 0.77 |
| Women | 0.02 (0.01) | 0.39 (0.36) | -0.59 (1.38) | 0.29 (0.25) | 0.06 (0.10) |  | 0.02 [0.00; 0.04] | 0%, P = 0.63 |
| Waist circumference change, cm/y | |  |  |  |  |  |  |  |
| BMI < 25 kg/m^2^ | 0.04 (0.01)* | -0.06 (0.33) | - | 0.06 (0.60) | 0.15 (0.11) |  | 0.04 [0.02; 0.06] | 0%, P = 0.78 |
| BMI ≥ 25 kg/m^2^ | 0.03 (0.01) | -0.50 (0.26) | -0.51 (1.05) | 0.34 (0.32) | 0.05 (0.05) |  | 0.03 [0.01; 0.05] | 28%, P = 0.24 |
| Men | 0.02 (0.01) | -0.47 (0.22) | -0.06 (1.37) | 0.42 (0.64) | 0.06 (0.05) |  | 0.02 [0.00; 0.04] | 33%, P = 0.20 |
| Women | 0.03 (0.02) | 0.02 (0.54) | -1.77 (1.78) | 0.30 (0.29) | 0.09 (0.13) |  | 0.03 [-0.01; 0.07] | 0%, P = 0.72 |
| *LNCB* |  |  |  |  |  |  |  |  |
| Body weight change, kg/y |  |  |  |  |  |  |  |  |
| BMI < 25 kg/m^2^ | 0.06 (0.01) | -0.12 (0.25) | - | 0.14 (0.59) | - |  | 0.06 [0.04; 0.08] | 0%, P = 0.76 |
| BMI ≥ 25 kg/m^2^ | 0.06 (0.01) | 0.18 (0.22) | -0.04 (0.53) | -0.45 (0.33) | - |  | 0.06 [0.04; 0.08] | 0%, P = 0.44 |
| Men | 0.07 (0.01) | 0.68 (0.28)* | 0.38 (1.59) | -0.78 (0.47)* | - |  | 0.08 [-0.57; 0.74] | 63%, P = 0.04 |
| Women | 0.06 (0.01) | -0.26 (0.19) | -0.12 (0.60) | -0.08 (0.35) | - |  | -0.03 [-0.24; 0.19] | 2%, P = 0.38 |
| Waist circumference change, cm/y | |  |  |  |  |  |  |  |
| BMI < 25 kg/m^2^ | 0.09 (0.01)* | -0.10 (0.40) | - | 0.14 (0.57) | - |  | 0.09 [0.07; 0.11] | 0%, P = 0.89 |
| BMI ≥ 25 kg/m^2^ | 0.10 (0.01) | 0.08 (0.26) | 0.08 (0.62) | -0.48 (0.36) | - |  | 0.10 [0.08; 0.12] | 0%, P = 0.44 |
| Men | 0.09 (0.01) | 0.71 (0.34) | 0.26 (1.36) | -0.82 (0.53) | - |  | 0.10 [-0.52; 0.72] | 52%, P = 0.10 |
| Women | 0.13 (0.01) | -0.34 (0.29) | 0.12 (0.78) | -0.04 (0.40) | - |  | 0.03 [-0.23; 0.30] | 0%, P = 0.42 |
| *the product term for the interaction was <0.05.  Models were adjusted for age, sex, and intervention group (Feel4diabetes, Predimed-Plus and AOC), baseline weight or waist circumference and height, education, physical activity, sedentary behaviour, alcohol intake, smoking; all dietary data, and total energy intake (only LNCB models).  AOC: Alpha Omega Cohort. BMI: Body Mass Index. CI: Confidence Interval; LNCB: Low/no-calorie beverage; SE: Standard Error; SSB: Sugar-sweetened beverage. | | | | | | | | |

Supplemental Table 8. Meta-analyses of the associations between the theoretical substitution of one serving beverage for another and yearly body weight and waist circumference change stratified by BMI and sex in the EU prospective cohort studies.

|  |  |  |  |  |  | Meta-analyses | |
| --- | --- | --- | --- | --- | --- | --- | --- |
| Models | Lifelines  β (SE) | NQplus  β (SE) | Predimed-Plus  β (SE) | Feel4Diabetes  β (SE) |  | Random-effects estimate  β (95%CI) | Heterogeneity  I^2^, P-value |
| *LNCB for SSB* | |  |  |  |  |  |  |
| Body weight change, kg/y |  |  |  |  |  |  |  |
| BMI < 25 kg/m^2^ | 0.02 (0.01) | -0.30 (0.32) | - | 0.52 (0.70) |  | 0.02 [0.00; 0.04] | 0%, P = 0.47 |
| BMI ≥ 25 kg/m^2^ | 0.06 (0.01) | 0.18 (0.31) | -0.59 (0.98) | -0.91 (0.42) |  | -0.14 [-0.63; 0.35] | 49%, P = 0.12 |
| Men | 0.05 (0.01) | 0.67 (0.33) | -0.43 (1.94) | -1.42 (0.69) |  | -0.08 [-1.00; 0.83] | 63%, P = 0.04 |
| Women | 0.04 (0.01) | -0.64 (0.41) | 0.46 (1.45) | -0.37 (0.42) |  | -0.15 [-0.55; 0.26] | 21%, P = 0.29 |
| Waist circumference change, cm/y | |  |  |  |  |  |  |
| BMI < 25 kg/m^2^ | 0.05 (0.02) | -0.04 (0.52) | - | 0.08 (0.72) |  | 0.05 [0.01; 0.09] | 0%, P = 0.98 |
| BMI ≥ 25 kg/m^2^ | 0.08 (0.02) | 0.59 (0.37) | 0.59 (1.15) | -0.82 (0.46) |  | 0.05 [-0.52; 0.62] | 49%, P = 0.12 |
| Men | 0.07 (0.01) | 1.19 (0.41) | 0.12 (1.72) | -1.24 (0.78) |  | 0.15 [-0.90; 1.20] | 71%, P = 0.02 |
| Women | 0.10 (0.02) | -0.35 (0.62) | 1.87 (1.87) | -0.34 (0.47) |  | 0.10 [0.06; 0.14] | 0%, P = 0.51 |
| *Water for SSB* | |  |  |  |  |  |  |
| Body weight change, kg/y |  |  |  |  |  |  |  |
| BMI < 25 kg/m^2^ | -0.02 (0.02) | 0.26 (0.37) | - | 0.36 (0.59) |  | -0.02 [-0.06; 0.02] | 0% , P = 0.61 |
| BMI ≥ 25 kg/m^2^ | -0.02 (0.02) | 0.36 (0.35) | -0.50 (1.09) | -0.47 (0.28) |  | -0.06 [-0.30; 0.19] | 24%, P = 0.27 |
| Men | -0.01 (0.02) | 0.37 (0.30) | -0.97 (1.87) | -0.75 (0.56) |  | -0.01 [-0.05; 0.03] | 17%, P = 0.31 |
| Women | -0.02 (0.02) | 0.15 (0.58) | 1.10 (1.73) | -0.28 (0.25) |  | -0.02 [-0.06; 0.02] | 0%, P = 0.66 |
| Waist circumference change, cm/y | | | | | | | |
| BMI < 25 kg/m^2^ | -0.01 (0.03) | 0.55 (0.61) | - | -0.06 (0.61) |  | -0.01 [-0.07; 0.05] | 0%, P = 0.65 |
| BMI ≥ 25 kg/m^2^ | -0.06 (0.03) | 0.98 (0.42) | 1.14 (1.44) | -0.35 (0.32) |  | 0.17 [-0.48; 0.82] | 61%, P = 0.05 |
| Men | -0.02 (0.02) | 1.18 (0.37) | -0.89 (1.60) | -0.47 (0.65) |  | 0.21 [-0.64; 1.06] | 73%, P = 0.01 |
| Women | -0.06 (0.03) | 0.29 (0.88) | 3.87 (2.46) | -0.29 (0.29) |  | -0.06 [-0.12; -0.00] | 10%, P = 0.34 |
| *Water for LNCB* | | | | | | | |
| Body weight change, kg/y |  |  |  |  |  |  |  |
| BMI < 25 kg/m^2^ | -0.05 (0.02) | 0.55 (0.40) | - | -0.08 (0.56) |  | 0.02 [-0.28; 0.33] | 11%, P = 0.33 |
| BMI ≥ 25 kg/m^2^ | -0.05 (0.02) | 0.26 (0.38) | 0.07 (0.59) | 0.48 (0.33) |  | 0.08 [-0.21; 0.37] | 8%, P = 0.35 |
| Men | -0.06 (0.02) | -0.25 (0.39) | 1.46 (3.15) | 0.80 (0.46) |  | 0.03 [-0.34;0.39] | 24%, P = 0.27 |
| Women | -0.04 (0.02) | 0.65 (0.42) | 0.08 (0.68) | 0.06 (0.35) |  | 0.01 [-0.20; 0.23] | 0%, P = 0.42 |
| Waist circumference change, cm/y | | | | | | | |
| BMI < 25 kg/m^2^ | -0.04 (0.03) | 0.60 (0.65) | - | -0.13 (0.58) |  | -0.04 [-0.10; 0.02] | 0%, P = 0.61 |
| BMI ≥ 25 kg/m^2^ | -0.08 (0.02) | 0.46 (0.45) | 0.33 (0.73) | 0.49 (0.37) |  | 0.14 [-0.25; 0.53] | 27%, P = 0.25 |
| Men | -0.06 (0.02) | 0.09 (0.49) | 1.52 (2.52) | 0.83 (0.53) |  | 0.10 [-0.33; 0.53] | 9%, P = 0.35 |
| Women | -0.10 (0.02) | 0.55 (0.64) | 0.51 (0.96) | 0.02 (0.40) |  | -0.10 [-0.14;-0.06] | 0%, P = 0.68 |
| Models were adjusted for age, sex, and intervention group (Feel4diabetes, Predimed-Plus), baseline weight or waist circumference and height, education, physical activity, sedentary behaviour, alcohol intake, smoking; all dietary data and total energy intake (only water for LNCB models).  BMI: Body Mass Index. CI: Confidence Interval. LNCB: Low/no-calorie beverage; SE: Standard Error. SSB: Sugar-sweetened beverage. | | | | | | | |

Supplemental Table 9. Sensitivity analyses of sugar-sweetened beverages (SSB) and low/no-calorie beverages (LNCB) with body weight and waist circumference change after excluding participants with history of diseases (type 2 diabetes, cardiovascular diseases, cancer, hypertension and/or hypercholesterolemia) ^a^.

|  |  |  |  |  |  | Meta-analyses | |
| --- | --- | --- | --- | --- | --- | --- | --- |
| Models |  | Lifelines  β (SE) | NQplus  β (SE) | Feel4Diabetes  β (SE) |  | Random-effects estimate  β (95%CI) | Heterogeneity  I^2^, P-value |
|  | N | 51,525 | 899 | 270 |  |  |  |
| *Body weight change, kg/y* |  |  |  |  |  |  |  |
| SSB |  | 0.02 (0.01) | 0.03 (0.17) | -0.50 (0.42) |  | 0.02 [-0.00;0.04] | 0%, P = 0.46 |
| LNCB |  | 0.05 (0.01) | 0.16 (0.20) | 0.38 (0.33) |  | 0.05 [0.03;0.07] | 0%, P = 0.52 |
| Substitution LNCB for SSB |  | 0.03 (0.01) | 0.13 (0.27) | 0.88 (0.50) |  | 0.04 [-0.03; 0.11] | 34%, P = 0.22 |
| Substitution water for SSB |  | -0.03 (0.02) | 0.18 (0.31) | 0.47 (0.42) |  | 0.00 [-0.16; 0.16] | 0%, P = 0.39 |
| Substitution water for LNCB |  | -0.05 (0.02) | 0.06 (0.35) | -0.37 (0.33) |  | -0.05 [-0.09;-0.01] | 0%, P = 0.60 |
|  |  |  |  |  |  |  |  |
| *Waist circumference chance, cm/y* |  |  |  |  |  |  |  |
| SSB |  | 0.03 (0.01) | -0.46 (0.23) | -0.88 (0.54) |  | -0.27 [-0.75; 0.20] | 73%, P = 0.03 |
| LNCB |  | 0.12 (0.01) | 0.12 (0.27) | 0.48 (0.43) |  | 0.12 [0.10; 0.14] | 0%, P = 0.70 |
| Substitution LNCB for SSB |  | 0.08 (0.01) | 0.59 (0.37) | 1.31 (0.64) |  | 0.44 [-0.18; 1.05] | 64%, P = 0.06 |
| Substitution water for SSB |  | -0.03 (0.02) | 0.85 (0.42) | 0.84 (0.53) |  | 0.41 [-0.25; 1.08] | 75%, P = 0.02 |
| Substitution water for LNCB |  | -0.08 (0.02) | 0.30 (0.48) | -0.47 (0.43) |  | -0.08 [-0.12; -0.04] | 0%, P = 0.48 |
| ^a^ In Lifelines and NQplus, participants were excluded if they self-reported type 2 diabetes at baseline, past or current history of cancer, cardiovascular diseases, hypertension or hypercholesterolemia. In Feel4diabetes, participants were excluded based on self-reported diabetes at baseline and/or diagnostics of hypertension or hypercholesterolemia at baseline. AOC and Predimed-Plus were not included as all participants had a history of one of these diseases at baseline.  Models were adjusted for age, sex, and intervention group (latter only in Feel4diabetes), baseline weight or waist circumference and height, education, physical activity, sedentary behaviour, alcohol intake, smoking; all dietary data, history of diabetes, hypertension, hypercholesterolemia, cardiovascular diseases and total energy intake (only for LNCB and water for LNCB replacement).Substitution models with water in Lifelines included N=14,907 when participants with history of diseases excluded.  LNCB: Low/no-calorie beverage; SE: Standard Error; SSB: Sugar-sweetened beverage. | | | | | | | |
